# SBFI Inhibitors Reprogram Transcriptomic Landscape of Prostate Cancer Cells Leading to Cell Death

**DOI:** 10.3390/cancers17233723

**Published:** 2025-11-21

**Authors:** Shubhra Rajput, Joseph F. LaComb, Chris Gordon, Hehe Wang, Manisha Sarder, Martin Kaczocha, Iwao Ojima, Agnieszka B. Bialkowska

**Affiliations:** 1 Department of Chemistry, Stony Brook University, Stony Brook, NY 11794-3400, USAiwao.ojima@stonybrook.edu (I.O.); 2 Department of Medicine, Renaissance School of Medicine, Stony Brook University, Stony Brook, NY 11794-8176, USA; 3 Department of Anesthesiology, Renaissance School of Medicine, Stony Brook University, Stony Brook, NY 11794-8480, USA; 4 Institute of Chemical Biology and Drug Discovery, Stony Brook University, Stony Brook, NY 11794-3400, USA; 5 Cancer Center, Stony Brook University, Stony Brook, NY 11794-8176, USA

**Keywords:** prostate cancer, FABP5, metastatic castration-resistant prostate cancer

## Abstract

Background: Prostate cancer (PCa) remains the second leading cause of cancer-related deaths in men in the United States. Fatty acid-binding protein 5 (FABP5), a member of a class of intracellular lipid transporters, promotes PCa progression via enhanced lipid metabolism and trafficking of lipid ligands. Previous work from our group has demonstrated that small-molecule FABP5 inhibitors based on the truxillic-acid monoester scaffold reduce PCa growth. Methods: Here, we assessed the effect of third-generation FABP5 inhibitors on the PCa cell cycle, proliferation, apoptosis, signaling pathway activity, and transcriptomic landscape. Results: We demonstrate that the third-generation FABP5 inhibitor SBFI-1143 significantly inhibits the viability of PCa cells by arresting them at the G0/G1 and G2/M phases of the cell cycle, inducing apoptosis, and promoting cell death. Strikingly, SBFI-1143 efficiently inhibited the growth of PCa spheroids compared to its predecessor, SBFI-103. RNA-seq and Gene Set Enrichment Analysis demonstrated that SBFI-1143 more effectively suppressed pathways involved in cell cycle progression, cell cycle division, and chromosome organization while upregulating genes associated with endoplasmic reticulum stress, responses to incorrectly folded proteins, and regulating apoptosis, compared to SBFI-103. Notably, SBFI-1143 treatment downregulated genes associated with the subpopulation of PCa cells characterized by a lineage plasticity-related signature, related to trans-differentiation, recurrence, and poor cancer prognosis. Conclusions: Our findings demonstrate that SBFI-1143 significantly alters the transcriptomic landscape of prostate cancer and may serve as a potentially effective therapeutic option for this disease.

## Simple Summary

Prostate cancer is the second leading cause of death in men, with the metastatic, castration-resistant cancer survival being under two years. This study aims to investigate the mechanism of action of a newly developed compound (SBFI-1143) targeting Fatty acid-binding protein 5 (FABP5), shown to play a critical role in prostate cancer development and progression. We demonstrated that SBFI-1143 is significantly more efficacious in inhibiting cell proliferation, increasing apoptosis, and leading to cell death compared to its precursors. Importantly, we showed, by employing RNA-seq and confirmatory analyses, that treatment with SBFI-1143 causes significant changes in the transcriptomics landscape of the prostate cancer cells, impacting pathways in cell cycle progression, cell division, and inducing cell stress. Our findings showed that SBFI-1143 can potentially serve as a therapeutic in treating metastatic, castration-resistant prostate cancer.

## 1. Introduction

Prostate cancer (PCa) is the most common cancer in men and cannot be averted by lifestyle changes or public health interventions, with incidence predicted to double by 2040 [1]. There is no available efficacious treatment for metastatic and castration-resistant prostate cancer (mPC and CRPC, respectively). The first originates through the evolution of the tumor, and the second is the unfortunate effect of the clinical regimen [2]. Current treatment of localized prostate cancer includes surgery and radiotherapy, while androgen deprivation therapy, androgen signaling inhibition, checkpoint and immune therapy, and chemotherapy are given during recurrent and metastatic disease [3]. Only one-third of the mPC patients will survive five years after diagnosis, and the median survival of CRPC patients is 1.86 years [4]. Thus, there is an unmet need to develop new therapies to improve the clinical outcome of the mPC and CRPC treatment.

PCa is associated with a limited somatic mutational burden, showing most changes in the gene copy number. Studies have identified multiple gene alterations such as gene fusions, hotspot mutations, loss of gene expression via promoter hypermethylation, or genetic instability leading to cancer initiation and progression [2]. The mPC and CRPC tumors have increased mutational burden and copy number alteration compared to the primary, localized disease. Recent progress in next-generation sequencing and an increased number of available biospecimens from patients from various disease stages allow for sophisticated analysis and more precise delineation of the disease progression. LaTulippe and colleagues determined that genes involved in cell cycle regulation, DNA replication and repair, transcriptional regulation, and cell structure and motility are characteristics of mPC [5]. Integrating data from RNA-seq and microarrays allowed for identifying genes involved explicitly in tumor initiation, progression, metastasis, and chemoresistant development [6,7]. Work from Sheng’s laboratory presented results of the integrative analysis that defined the cell-intrinsic signature of neuroendocrine prostate cancer and lineage plasticity-related signature (LPSig). Furthermore, they identified a subpopulation of prostate cancer cells (LPCs) with a role in trans-differentiation, recurrence, and poor prognosis and provided their genetic signature [8,9]. Moreover, dysregulation of lipid metabolism, lipid signaling pathways, and alteration to oxidative phosphorylation were shown as prominent characteristics of prostate cancer [10,11,12,13,14,15].

Fatty acid-binding proteins (FABPs) are a family of ten members of intracellular lipid-binding proteins regulating lipids’ transport and cellular metabolism [16,17,18]. It has been previously shown that FABP5 regulates multiple processes, such as lipid metabolism, proliferation, differentiation, inflammation, and PCa development [19,20,21,22,23]. Recently, our group showed that FABP5 has high copy number alteration in PCa with the highest expression among all FABPs, which is highly correlated to the *MYC* gene amplification [24]. Consistently increased levels of FABP5 have been shown in mCRPC resistant to taxanes [7]. In contrast, deleting *FABP5* generated similar consequences as deleting the androgen receptor (*AR*) in prostate cancer cells, a major driver of the disease progression [25]. In addition, it has been demonstrated that *FABP5* inactivation suppresses PCa growth and sensitizes prostate cancer cells to drugs targeting microtubules, leading to cell death [26,27].

Recent compelling publications from us and others demonstrate that FABP5 inhibitors based on the truxillic-acid monoester (TAMEs) scaffold have a profound effect on PCa progression [19,24,26,28,29,30]. These compounds potently inhibit PCa development and progression by downregulating components of crucial pathways readily utilized by PCa towards its progression and metastasis. We have demonstrated that SBFI-103 (a 2nd-generation compound) has a strong inhibitory effect in multiple prostate cancer cell lines and exerts potent inhibitory effects on metastatic and castration-resistant PCa [24], two significant hurdles that any treatment has not yet overcome.

Here, we present the first in-depth study analyzing the mechanism of SBFI compounds’ action in an in vitro PCa model. Treatment with SBFI-1143 caused alterations to the cell cycle, leading to potent decreased viability and proliferation and resulting in cell death in a dose- and time-dependent manner compared to its predecessors. Transcriptomic analysis showed vast downregulation of genes associated with cell cycle, cell division, and chromosome organization. Significantly, SBFI-1143 treatment reduced the levels of almost all signature genes, marking the subpopulation of PCa cells responsible for trans-differentiation, recurrence, and poor cancer prognosis. In summary, our study demonstrates the broad efficacy of SBFI-1143 against PCa in vitro and supports its translational potential.

## 2. Materials and Methods

### 2.1. Cell Lines and Reagents

Human prostate cancer cell lines PC-3 (ATCC, CRL-1435, RRID:CVCL_0035) and DU 145 (ATCC, HTB-81, RRID:CVCL_0105) were purchased from ATCC, and mouse prostate cancer cell line RCaP was a gift from the laboratory of Dr. Lloyd Trotman. PC-3 and RCaP cell lines were cultured in RPMI-1640 medium (Corning, Oneonta, NY, USA, 10-040-CV) and DU 145 in Eagle’s Minimum Essential Medium (Corning, 10-009-CV). All media were supplemented with 10% FBS (Peak Serum, Bradenton, FL, USA, PS-FB3) and 1% antibiotic/antimycotic (10,000 U/mL penicillin and 10,000 µg/mL streptomycin; Thermo Fisher Scientific, Waltham, MA, USA, 15140-122) and maintained in a humidified incubator with 5% CO_2_ at 37 °C. The cells were routinely assessed for *Mycoplasma* contamination and cells passaged less than 25 times were used for the in vitro experiments. Dimethylsulfoxide (DMSO; Thermo Fisher Scientific, Waltham, MA, USA, BP231-100) was used as a vehicle control at a final 0.1–0.2% concentration. First-, second-, and third-generation TAMEs, SBFI-26, SBFI-103, and SBFI-1143, previously referred to as L1, L3, and α-4, respectively, were synthesized and characterized as previously discussed [29]. SBFI-26 and 103 were reconstituted in DMSO at a final concentration of 100 mmol/L, and SBFI-1143 was reconstituted at a final concentration of 50 mmol/L. All the drug stocks were stored at −20 °C until further use.

### 2.2. Cell Proliferation

For the 2D system, PC-3 cells were seeded (10^5^ cells/well) in six-well flat bottom plates (USA Scientific, Ocala, FL, USA, CC7682-7506) in 2 mL of appropriate media and incubated for 24 h. Following incubation, the cell media was aspirated and replaced with the growth media supplemented with vehicle control (0.2% DMSO) or various concentrations of SBFI-26, SBFI-103, and SBFI-1143 (5, 10, 15, 20, 50, and 100 μmol/L). The cells were collected after 24, 48, and 72 h of treatment and counted using the Z-Series Coulter Counter (Beckman Coulter, Brea, CA, USA). The measurement of the control (cells with 0.2% DMSO) was defined as 100%, and treatment measurements were calculated accordingly.

For the spheroid (3D) model, PC-3 cells were seeded (2500 cells/well) in a 96-well round bottom ultra-low attachment surface spheroid microplate (Corning, 4520) in 50 μL of appropriate media and incubated for 24 h. Following incubation, the growth media were supplemented with 50 μL vehicle control (0.2% DMSO) or SBFI-103 and SBFI-1143 with a final concentration of 15 μmol/L. Images of the spheroids were taken 24, 48, and 72 h post-treatment using the Nikon ECLIPSE Ti2 inverted microscope (Nikon, Brighton, MI, USA, RRID:SCR_021068). The cell viability was assessed 72 h after the treatment using 3D Cell Titer-Glo (Promega, Madison, WI, USA, G9683) and measured on the SpectraMax M3 Microplate Reader (Molecular Devices, San Jose, CA, USA).

### 2.3. Cell Cycle Analysis

PC-3 cells were seeded (10^5^ cells/well) in six-well flat bottom plates in 2 mL of appropriate media and incubated for 24 h. Following incubation, the cell media was aspirated and replaced with the growth media supplemented with vehicle control (0.2% DMSO) or various concentrations of SBFI-26, SBFI-103, and SBFI-1143 (5, 10, 15, 20, 50, and 100 μmol/L). The cells were collected after 24, 48, and 72 h of treatment and fixed overnight in 70% ethanol diluted in Dulbecco’s PBS (1x DPBS; Corning, 21-031-CV). The cells were stained with 2 mmol/L of propidium iodide and analyzed using the CytoFLEX Flow Cytometer (Beckman Coulter, RRID:SCR_025067). The data were analyzed using the software Modfit (https://www.vsh.com/products/mflt/ (accessed on 26 May 2023), RRID:SCR_016106).

### 2.4. Apoptosis Assay

PC-3 cells were seeded (10^5^ cells/well) in six-well flat bottom plates in 2 mL of appropriate media and incubated for 24 h. Following incubation, the cell media was aspirated and replaced with the growth media supplemented with vehicle control (0.2% DMSO) or various concentrations of SBFI-26, SBFI-103, and SBFI-1143 (5, 10, 15, 20, 50, and 100 μmol/L). The cells were collected after 24, 48, and 72 h of treatment. After collecting, the cell pellet was resuspended in 1X Annexin-binding buffer and stained with FITC Annexin V and propidium iodide (1.5 mmol/L) using the Dead Cell Apoptosis Kit (Thermo Fisher Scientific, Waltham, MA, USA, V13242). The cells were immediately analyzed using the CytoFLEX Flow Cytometer (Beckman Coulter), and fluorescence emission was measured at 530 nm and >575 nm.

### 2.5. RNA-Sequencing

For RNA-sequencing analysis, PC-3 and RCaP cells (6 × 10^5^ cells/well) were seeded in 100 mm flat bottom clear plates (Thermo Fisher Scientific, Waltham, MA, USA, 08-772E) in 10 mL of appropriate media and incubated for 24 h. The media was aspirated after incubation and replaced with fresh growth media supplemented with negative control (0.2% DMSO) or 15 μmol/L SBFI-103 or SBFI-1143. Total RNA was isolated after 24 and 48 h of treatment using the QIAGEN RNeasy Mini Kit (QIAGEN, Germantown, MD, USA, 74104). All the RNA samples were measured using High Sensitivity RNA TapeStation Assay (Agilent Technologies, Santa Clara, CA, USA, TapeStation 4200, RRID:SCR_018435) for their integrity and delivered an RNA integrity number (RIN) value of >9. Novogene Corporation Inc. (Sacramento, CA, USA) performed the RNA-seq. The pre- and post-alignment quality controls, alignment to the reference human genome using HISAT2 [31] (hg38 and mm10), and transcripts’ quantification with featureCounts [32] were performed on https://usegalaxy.org/ (accessed on 23 April 2024 and 5 May 2024). Normalization, differential gene expression, and pathway analysis were performed in R version 4.2.2 using DESeq2 [33] with vsd and apeglm [34] (data transformation) and topVarGene (gene clustering). Genes with an adjusted *p* < 0.05 and at least 2-fold change were selected as differentially expressed genes (DEGs) processed with Enhanced Volcano and pheatmap (data visualization), Gene Set Enrichment Analysis (GSEA) [35], and Gene Ontology [36] (pathway analysis). The data are deposited at NCBI GEO platform (GSE297940, GSE297941, and GSE309891).

### 2.6. Quantitative Reverse Transcriptase Polymerase Chain Reaction (RT-qPCR)

PC-3, DU 145, and RCaP cells (6 × 10^5^ cells/well) were seeded in 100 mm flat bottom clear plates (Falcon, 08-772E) in 10 mL of appropriate media and incubated for 24 h. The media was aspirated after incubation and replaced with fresh growth media supplemented with negative control (0.2% DMSO) or 15 μmol/L SBFI-103 and SBFI-1143. Total RNA was isolated after 24 h of treatment using the QIAGEN RNeasy Mini Kit (QIAGEN, 74104), followed by cDNA preparation using SuperScript IV VILO Master Mix (Invitrogen, 11756050). *ACTB* (ID: Hs01060665_g1, 4448489) and *Actb* (ID: Mm04394036_g1, 4448485) were used as housekeeping genes. TaqMan primers used were as follows: *CDK1* (ID: Hs00938777_m1, 4453320), *CDK2* (ID: Hs01548894_m1, 4453320), *CCNA2* (ID: Hs00996788_m1, 4453320), *CCNB1* (ID: Hs01030099_m1, 4453320), *CCND1* (ID: Hs00765553_m1, 4453320), *CENPA* (ID: Hs00156455_m1, 4453320), *ZNF695* (ID: Hs00737185_m1, 4448892), *ZNF367* (ID: Hs01572698_m1, 4448892), *E2F7* (ID: Hs00987777_m1, 4448892), *FOXM1* (ID: Hs01073586_m1, 4453320), *Cdk1* (ID: Mm00772472_m1, 4453320), *Cdk2* (ID: Mm00443947_m1, 4453320), *Ccna2* (ID: Mm00438063_m1, 4453320), *Ccnb1* (ID: Mm00838401_g1, 4453320), *Ccnd1* (ID: Mm00432359_m1, 4453320), *Cenpa* (ID: Mm00483253_g1, 4448892), *Zpf367* (ID: Mm00615562_m1, 4448892), *E2f7* (ID: Mm00618098_m1, 4448892), and *Foxm1* (ID: Mm00514924_m1, 4453320). RT-qPCR quantification was performed using the QuantStudio 3 Real-Time PCR System (Applied Biosystems, Waltham, MA, USA, A28567, RRID:SCR_018712). The 2^−ΔΔCt^ method [37] was used to determine the relative gene expression.

### 2.7. ChIP-X Enrichment Analysis 3 (ChEA3)

The transcriptional factors enrichment analysis was performed using ChEA3 software, version 3 [38]. The list of significantly downregulated and upregulated genes with an adjusted *p* < 0.05 and at least 2-fold change were inputted into https://maayanlab.cloud/chea3/ (accessed on 30 April 2024) to retrieve the list of transcription factors.

### 2.8. Western Blot Analysis

PC-3, DU 145, and RCaP cells (6 × 10^5^ cells/well) were seeded in 100 mm flat bottom clear plates in 10 mL of appropriate media and incubated for 24 h. The media was aspirated after incubation and replaced with fresh growth media supplemented with negative control (0.2% DMSO) or 15 μmol/L SBFI-103 or SBFI-1143. The cells were collected for protein after 24 h of treatment in 2x Laemmli buffer, and total protein extracts were subjected to electrophoresis in 4–20% Tris-HCl gels (Bio-Rad Laboratories, Hercules, CA, USA, 5671093). The proteins were transferred to a nitrocellulose membrane followed by blocking in 5% *w*/*v* non-fat dry milk in 1x TBST. The membranes were washed twice after the blocking with 1x TBST and incubated overnight in the respective primary antibodies at 4 °C. The membranes were washed the following day in 1x TBST and incubated with HRP-conjugated secondary antibodies at room temperature for 1 h. Signals were detected using SuperSignal West Pico PLUS Chemiluminescent Substrate (Thermo Fisher Scientific, Waltham, MA, USA, 34578) and captured with the Azure 400 Western Blot Imager (Azure Biosystems, Dublin, CA, USA, 10147-218). Antibodies and dilutions were as follows: anti-CDK2 (Cell Signaling Technology, Danvers, MA, USA, 18048, RRID:AB_2923174, 1:1000 in BB), anti-CDC2 (Santa Cruz Biotechnology, Dallas, TX, USA, sc-54, RRID:AB_627224 1:1000 in BB), anti-Cyclin A2 (Cell signaling Technology, 67955, RRID:AB_2909603 1:1000 in BSA), anti-Cyclin B1 (Cell Signaling Technology, 12231, RRID:AB_2783553, 1:1000 in BSA), anti-Cyclin D1 (BioCare Medical, Pacheco, CA, USA, CRM307A, 1:1000 in BSA), and anti–β-Actin (Millipore Sigma, Burlington, MA, USA, A1978, RRID: AB_476692, 1:2000 in BB). Densitometry was analyzed using the Gel analyzer function on ImageJ version 1.53i, [39] (RRID: SCR_003070; ref. [36]) and normalized to β-Actin control.

### 2.9. Statistical Analysis

Statistical significance (*p* < 0.05) was determined using two-way ANOVA with the Tukey test for multiple comparisons and Student’s *t*-test for two comparison groups. The experiments were performed in triplicate. All statistical analyses were performed using GraphPad Prism (RRID: SCR_002798) software version 10.0.2.

## 3. Results

### 3.1. The Third-Generation FABP5 Inhibitor (SBFI-1143) Significantly Reduced the Proliferation of Prostate Cancer Cells

**Figure 1:**
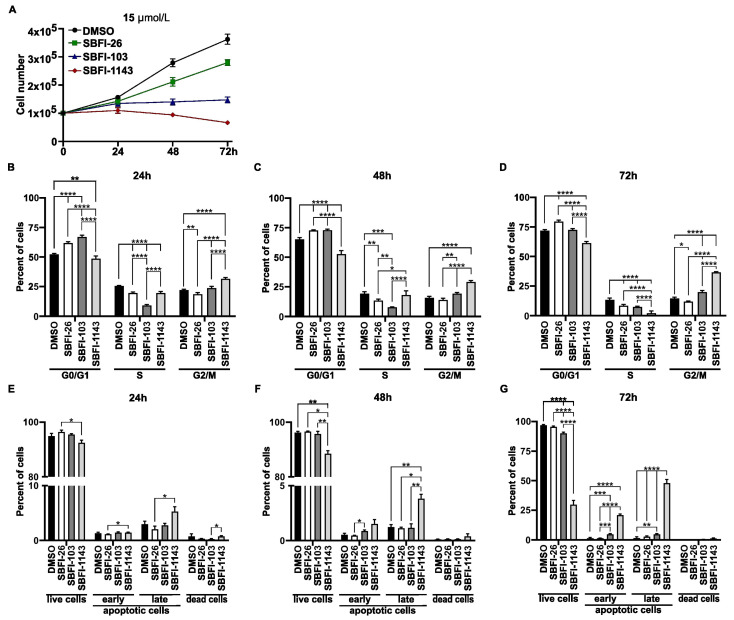
The third-generation inhibitor, SBFI-1143, significantly reduces proliferation, alters cell cycle progression, and induces apoptosis compared to controls. PC-3 cells were treated with a 15 μmol/L concentration of SBFI-26, SBFI-103, or SBFI-1143 for 24, 48, and 72 h. (**A**) Cell proliferation. Cells were counted using a cell counter at each time point, and data represents mean ± SD (N = 3). The measurement of the control (0.2% DMSO) was defined as 100%. (**B**–**D**) Cell cycle progression was assessed by combining PI stain and flow cytometry. Data represents mean ±SD with N = 3. Two-way ANOVA with Tukey’s multiple range test significance is shown as * *p* < 0.05; ** *p* < 0.01; *** *p* < 0.001; **** *p* < 0.0001. (**E**–**G**) The level of apoptosis was determined using Annexin V/PI stain with flow cytometry. Data are represented as mean ± SD (N = 3). Two-way ANOVA with Tukey’s multiple range test significance is shown as * *p* < 0.05; ** *p* < 0.01; *** *p* < 0.001; **** *p* < 0.0001. Panels (**D**) and (**G**) previously shown in [29] and modified.

Currently, we developed the third-generation inhibitor, SBFI-1143, that showed markedly increased efficacy in inhibiting PCa growth [29]. Our recent publication touched upon the stronger efficacy of the SBFI-1143 in modifying the cell cycle and induction of apoptosis of PCa cells after three days of treatment compared to first- and second-generation SBFI compounds. However, the broad mechanism of action and effect on the transcriptomic landscape of SBFI-1143 has not been established. To establish the conditions for assessing these changes, we first compared its effectiveness in inhibiting PCa cell growth. PC-3 cells were treated with various concentrations of first-, second-, and third-generation inhibitors: SBFI-26, SBFI-103, and SBFI-1143, respectively, and DMSO (vehicle) as a control. The cells were collected and counted 24, 48, and 72 h post-treatment. The results demonstrated that SBFI-1143 has a more substantial inhibitory effect on prostate cell proliferation than SBFI-26 and SBFI-103 (Figure 1A and Supplementary Figure S1). We observed that at the 5 μmol/L concentration after two days of treatment, SBFI-1143 significantly and strongly inhibited the proliferation of PC-3 cells compared to other conditions (Supplementary Figure S1A). Its impact on cell growth was evident at 10 μmol/L, 15 μmol/L, and 20 μmol/L at 24 h post-treatment and persisting until the end of the treatment (Figure 1A and Supplementary Figure S1B,C). Only at 50 μmol/L and 100 μmol/L concentrations we observed a more potent effect of SBFI-103 than SBFI-1143 (Supplementary Figure S1D,E). However, both SBFIs at these ranges inhibited cell proliferation by 80–95%. In summary, SBFI-1143 potently reduces cell proliferation of PCa cells.

### 3.2. SBFI-1143 Alters the Cell Cycle Progression and Induces Cell Death of PCa Cells

Multiple factors, which result in changes in the cell cycle progression and induction of apoptosis, can cause reduction in cell proliferation. To assess whether SBFI-1143 affects the progression of the cell cycle, the impact of SBFI-26, SBFI-103, and SBFI-1143 was evaluated on prostate cancer cells. PC-3 cells were treated as described in the Material and Methods section and then the cell cycle was analyzed using PI stain combined with flow cytometry. We observed that all three tested compounds altered the cell cycle progression. The range of concentrations between 5 μmol/L and 20 μmol/L of SBFI-26 resulted in an increasing number of cells in the G0/G1 phase and reduction in the S-phase and G2/M phases throughout the treatment, compared to the control (Figure 1B–D, Supplementary Figures S2A–C, S3A–C, S4A–C and S5A–C). In the same concentration range, SBFI-103 and SBFI-1143 at tested time points caused a decrease in the cell number in the G0/G1 phase and increased G2/M while showing variable modifications to the S-phase. The comparison between SBFI-1143 and two other compounds shows that the alterations made by SBFI-1143 were significantly higher than those by SBFI-26 and SBFI-103 (Figure 1B–D and Supplementary Figures S2A–C, S3A–C, S4A–C and S5A–C). As shown in Figure 1, SBFI-103 more potently reduced cell proliferation of PC-3 cells than SBFI-1143 at 50 μmol/L and 100 μmol/L. This observation agrees with the results from the cell cycle analysis (Supplementary Figures S2D,E, S3D,E and S4D,E). SBFI-103 more efficiently reduced the number of cells in the S-phase and G2/M phases compared to SBFI-1143. However, SBFI-1143 still caused a reduction in cells in the G0/G1 phases and an increase in the S-phase and G2/M. In summary, while all three SBFIs alter the progression of the cell cycle, SBFI-1143 is more potent among tested compounds, particularly at lower concentrations.

The reduction in cell proliferation and alterations to the cell cycle caused by SBFIs may result in the arrest of proliferation and lead to apoptosis and cell death. To determine whether SBFI-26, SBFI-103, and SBFI-1143 are cytotoxic and cause cell death, PC-3 were treated with various concentrations of these SBFIs over three days and Annexin V/PI stain and flow cytometry were performed. The results showed that 5 μmol/L and 10 μmol/L concentrations of SBFIs did not induce cell death in PC-3 cells (Supplementary Figures S6A,B, S7A,B and S8A,B). However, 24 h after treatment, SBFI-1143 at 15 μmol/L and 20 μmol/L already significantly reduced the population of healthy cells and increased the cells in early and late apoptosis and cell death (Figure 1E–G and Supplementary Figures S6C, S7C, S8C and S9A–C). At the same time, SBFI-103 causes a similar effect only at 20 μmol/L. Notably, at 15 μmol/L and 20 μmol/L, SBFI-1143 most efficiently reduced viable cell population, increased early and late apoptosis, and induced cell death compared to other controls (Figure 1E–G and Supplementary Figures S6C, S7C, S8C and S9A–C). At high concentrations of 50 μmol/L and 100 μmol/L, both SBFI-103 and SBFI-1143 eliminated almost all viable cells with significant increases in early and late apoptosis, with SBFI-1143 being significantly more efficacious compared to SBFI-26 and even SBFI-103 (Supplementary Figures S6D,E, S7D,E and S8D,E).

**Figure 2:**
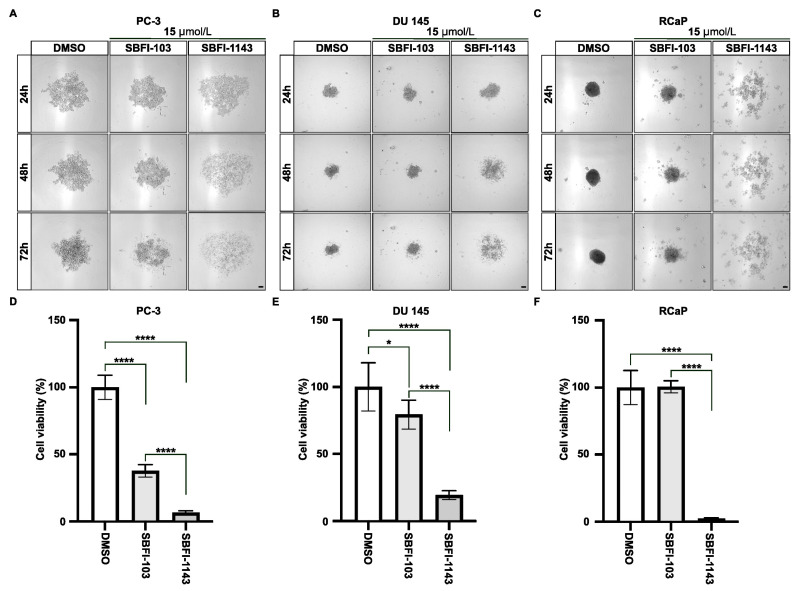
The third-generation inhibitor, SBFI-1143, significantly reduces viability of PCa spheroids. (**A**–**C**) The representative images of PC-3, DU 145, and RCaP cells, respectively, grown as spheroids and treated for 24, 48, and 72 h with DMSO, 15 μmol/L of SBFI-103, and 15 μmol/L of SBFI-1143. Scale bar represents 100 μm. (**D**–**F**) The viability of spheroids (**A**–**C**) was assessed using 3D CTG Glo with DMSO, which was defined as 100%. Data represents mean ± SD with N = 6. Two-way ANOVA with Tukey’s multiple range test significance is shown as * *p* < 0.05, **** *p* < 0.0001.

Furthermore, we compared the ability of SBFI-103 and SBFI-1143 to inhibit the proliferation of PCa cells grown in three-dimensional culture as spheroids. The results demonstrated that 15 μmol/L SBFI-1143 significantly reduced the viability of spheroids of PC-3, DU 145, and RCaP cells compared to control and to treatment with 15 μmol/L SBFI-103 (Figure 2A–F). These results show that all three compounds can induce apoptosis and cell death. However, the third-generation inhibitor SBFI-1143 was the most effective.

### 3.3. SBFI-103 and SBFI-1143 Modify the Transcriptome of PCa Cells

To comprehensively assess the impact of SBFI-103 and SBFI-1143 on the gene expression of prostate cancer cells, RNA-seq analysis was performed. Based on the gathered cell proliferation, cell cycle, and apoptosis data (Figure 1 and Supplementary Figures S1–S9), significant reductions in proliferation, modifications to the cell cycle, and an increase in apoptosis were observed as early as 24 h. Thus, a 15 μmol/L concentration of the SBFI-103 and SBFI-1143 was selected for RNA-seq. Three sets of experiments treating PC-3 cells with DMSO (vehicle) and 15 μmol/L concentration of SBFI-103 and SBFI-1143 for 24 h, PC-3 cells with DMSO (vehicle) and 15 μmol/L SBFI-1143 for 48 h, and RCaP cells with DMSO (vehicle) and 15 μmol/L concentration of SBFI-103 and SBFI-1143 for 24 h were carried out, and appropriate analyses were performed.

We followed stringent significance guidelines, only including functional genes with 95% confidence and an adjusted *p* value of <0.05. We first conducted principal component analysis (PCA) (Supplementary Figures S10A and S11A) and proximity gene analysis shown as a sample-to-sample distance heatmap (Supplementary Figures S10B and S11B) of DMSO-treated and SBFI-103- and SBFI–1143-treated PC-3 and RCaP cells for 24 h, respectively. These analyses confirmed a strong correlation within biological replicates and revealed differences in clustering between treatment conditions. We employed gene clustering and the TopVarGenes function to identify the top 20 differentially expressed genes between SBFI treatments and DMSO (Supplementary Figures S10C and S11C). Some of the most notable genes that were upregulated in PC-3 cells, such as Heme oxygenase 1 (*HMOX1*), Activating Transcription Factor 3 (*ATF3*), Interleukin 1 Receptor Type 1 (*ILIR1*), Niban apoptosis regulator 1 (*NIBAN1*), Sestrin-2 (*SESN2*), DNA damage-inducible transcript 4 (*DDIT4*), and ChaC glutathione specific γ-glutamyl cyclotransferase 1 (*CHAC1*), belong to stress response machinery. At the same time, highly downregulated genes, such as the RNA component of 7SK nuclear ribonucleoprotein (*RN7SK*), FAM111 trypsin-like peptidase B (*FAM111B*), Phosphatase and actin regulator 3 (*PHACTR3*), Ribonucleotide reductase regulatory subunit M2 (*RRM2*), Adhesion G protein-coupled receptor F1 (*ADGRF1*), Hyaluronan synthase 3 (*HAS3*), Mitochondrially encoded cytochrome c oxidase II (*COX2*), and DNA topoisomerase II alpha (*TOP2A*), are involved in positive regulation of cell proliferation, cell cycle progression, and tumor growth. Similar analysis of transcriptomic changes in RCaP cells showed upregulation of *Atf3* and significant downregulation of genes involved in cell growth, survival, and development, such as *Sfrp2*, *Aspn*, *Agtr2*, *Rspo2*, *Gas1* and *6*, *Tmem119*, *Efemp1*, and *Crabp1*, and extracellular matrix formation, migration, and metastasis, such as *Dpt*, *Igfbp5*, *Srpx*, *Col3a1*, *Col1a1*, *Cd248*, *S100a4*, *Ptn*, and *Nid2* (Supplementary Figure S11C). It should be noted that these genes’ levels of upregulation and downregulation are stronger upon SBFI-1143 treatment than SBFI-103.

**Figure 3:**
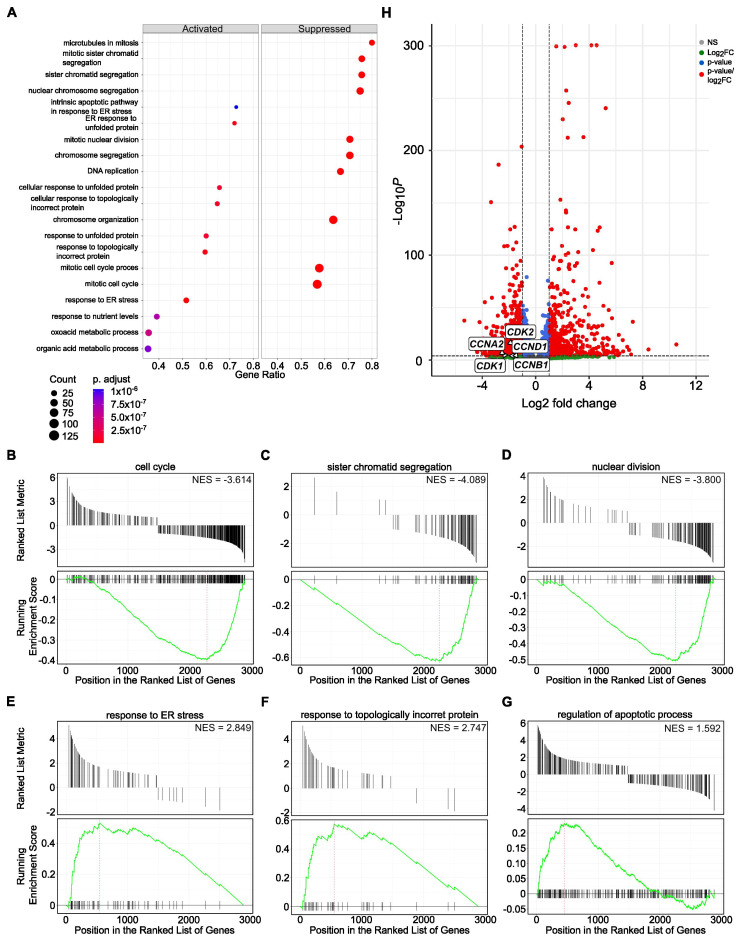
Functional enrichment of activated and suppressed genes and pathways upon SBFI-103 and SBFI-1143 treatment of PC-3 cells. (**A**) The top 10 activated and suppressed pathways were identified using gene ontology analysis using a cluster profiler. (**B**–**G**) Gene Set Enrichment Analysis (GSEA) individual gene set enrichment plots of selected three highly downregulated (**B**–**D**) and three highly upregulated (**E**–**G**) pathways in SBFI-treated samples compared to DMSO-treated. NES–normalized enrichment score. (**H**) Enhanced Volcano plot showing five downregulated transcripts of *CDK1*, *CDK2*, *CCNA2*, *CCNB1*, and *CCND1*. Red circles represent genes with |log_2_FC| ≥ 1 and adjusted *p* < 0.05, blue represents genes with adjusted *p* < 0.05 only, and gray represents genes that were neither eligible in conditions of adjusted *p*-value nor |log 2 FC|. Original western blots are presented in Supplementary Materials.

Further, we performed cumulative pathway analysis and presented data of the top 10 activated and suppressed pathways after treatment with both SBFIs compared to DMSO-treated PC-3 cells. The results demonstrated that the most suppressed pathways are related to the mitotic cell cycle, chromosome organization and segregation, DNA replication, and microtubule function in mitosis. In contrast, organic acid metabolic and oxoacid metabolic processes and cell stress responses are induced upon treatment (Figure 3A). Gene Set Enrichment Analysis (GSEA) confirmed pathways identified with the Gene Ontology, such as cell cycle, sister chromatid segregation, nuclear division, response to ER stress, and topologically incorrect protein or regulation of apoptotic process, to name a few (Figure 3B–G). Similar analysis in RCaP cells revealed significant downregulation of signaling pathways involved in extracellular matrix regulation and slight activation of pathways involved in stress and immune responses (Supplementary Figure S12A–G). To further analyze data from RNA-seq of PC-3 and RCaP cells, we interrogated significantly upregulated and downregulated genes in both datasets. The results showed that there are only a few upregulated genes, mainly belonging to the reactivation of the EGFR signaling pathway (Supplementary Figure S13A). In contrast, there are multiple downregulated pathways identified in the PC-3 and RCaP dataset (Supplementary Figure S13B). The components of commonly downregulated pathways regulate cell cycle progression (pro-metaphase, metaphase, anaphase), cell cycle checkpoints, kinetochores formation, and separation, and mitotic spindle formation.

**Figure 4:**
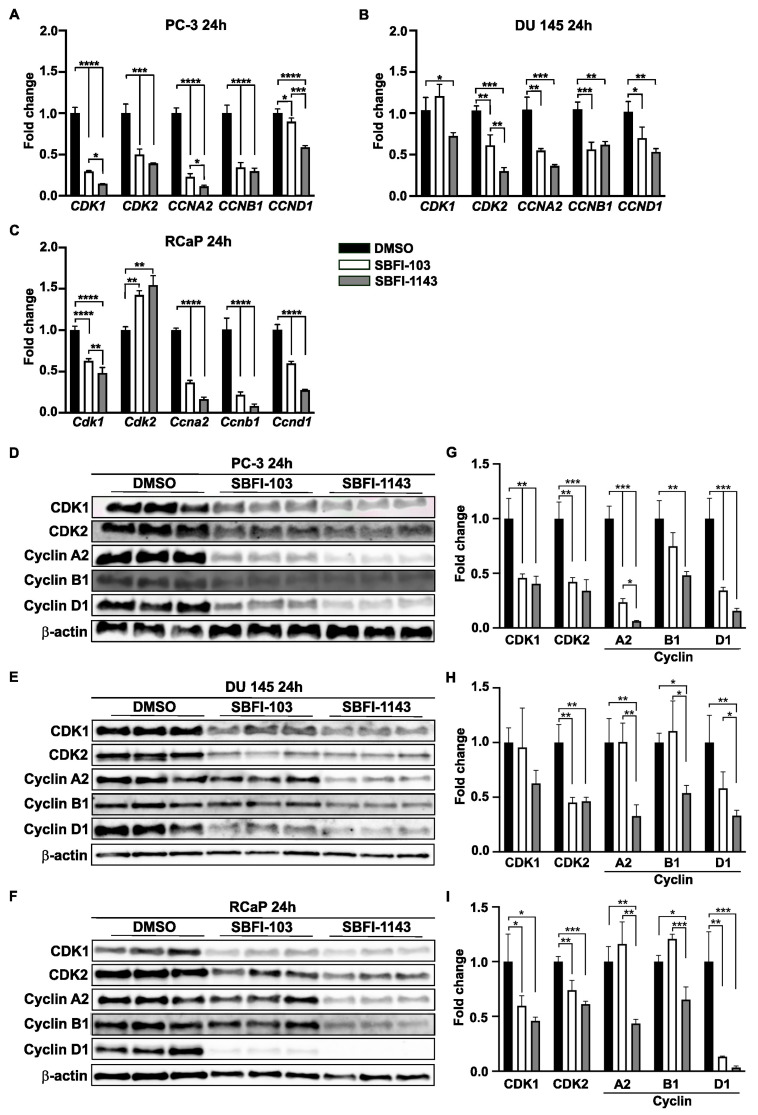
SBFIs decrease the expression of essential genes and proteins in cell cycle progression. PC-3, DU 145, and RCaP cells were treated with DMSO (control) and 15 μmol/L of SBFI-103 or SBFI-1143 for 24 h, and RNA and protein were collected for analysis. (**A**–**C**) qRT-PCR analysis of genes involved in cell cycle progression: *CDK1*, *CDK2*, *CCNA2*, *CCNB1*, and *CCND1* of PC-3, DU 145, and RCaP cells, respectively. (**D**–**I**) Immunoblotting and densitometry analysis of five essential cell cycle proteins CDK1, CDK2, Cyclin A1, Cyclin B1, and Cyclin D1. (**D**,**G**)—PC-3, (**E**,**H**)—DU 145, and (**F**,**I**)—RCaP. Data are represented as mean ± SD (N = 3). One-way ANOVA with Tukey’s multiple range test significance is shown as * *p* < 0.05; ** *p* < 0.01; *** *p* < 0.001; **** *p* < 0.0001.

Until now, the results showed that both SBFI-103 and SBFI-1143 negatively regulated cell proliferation, induced apoptosis, and altered cell cycle progression. RNA-seq analysis confirmed that cell cycle and DNA replications are among the top suppressed pathways in these SBFIs’ treatment compared to the control. Thus, it was decided to evaluate several genes involved in cell cycle progression based on the results of RNA-seq analysis. For the confirmatory RT-qPCR analysis five genes were selected, i.e., Cyclin-Dependent Kinase 1 (*CDK1*), Cyclin-Dependent Kinase 2 (*CDK2*), Cyclin A2 (*CCNA2*), Cyclin B1 (*CCNB1*), and Cyclin D1 (*CCND1*), as the proteins encoded by these genes have been shown to regulate G2/M, G1/S, and S/G2 transitions, which are the phases altered by SBFIs treatments. First, using the Enhanced Volcano feature, the results demonstrated that the levels of all five genes were significantly reduced upon SBFI-103 and SBFI-1143 treatment compared to the DMSO-treated PC-3 and RCaP cells (Figure 3H, Supplementary Figure S12H, and Supplementary Table S1, respectively). Hence, PC-3, DU 145, and RCaP cells with a 15 μmol/L concentration of SBFI-103, SBFI-1143, and DMSO were treated for 24 h and collected for RNA and protein analyses. The confirmatory RT-qPCR analysis showed that all selected genes were significantly downregulated upon treatment with both SBFIs in PC-3, DU 145, and RCaP PCa cells (Figure 4A–C). Interestingly, SBFI-1143 exhibited a more pronounced effect on all tested transcript levels than SBFI-103. Western blot analysis of PC-3, DU 145, and RCaP cells showed that the protein levels of all five genes are significantly decreased upon treatment with SBFIs compared to the DMSO-treated cells, and the levels of Cyclin A2 and Cyclin D1 are additionally more reduced upon SBFI-1143 treatment compared to SBFI-103 (Figure 4D–I). While the transcript level of *Cdk2* in RCaP cells increases upon SBFI-103 and SBFI-1143 treatment, the CDK2 protein levels are significantly decreased, which may suggest a compensatory or alternative mechanism of these compounds’ action in RCaP cells compared to the other two cell lines. Importantly, the reduction at the protein level was confirmed in several metastatic and/or castration-resistant prostate cancer cell lines such as DU 145 and RCaP, demonstrating a potentially universal response to the SBFI compound treatment (Figure 4D–I).

### 3.4. SBFI-1143 Alters Transcriptional Machinery and Impacts the Expression of Genes Involved in the Progression of mPC

The transcriptomic analysis and bioassays have demonstrated that SBFI-1143 has higher potency in inhibiting the growth of PCa cells. To further characterize the mechanism of action of SBFI-1143, we assessed changes to the transcriptome of PCa cells at different time points after the treatment. Therefore, PC-3 cells with a 15 μmol/L concentration of SBFI-1143 were treated for 24 and 48 h and RNA-seq analysis was performed. PCA and proximity gene analysis strongly correlated with biological replicates and differences between DMSO and SBFI-1143 while showing similarity between 24 and 48 h of SBFI-1143 treatments (Supplementary Figure S14A,B). Gene clustering analysis identified the top 20 differentially expressed genes between conditions and showed high similarity in response between 24 and 48 h after SBFI-1143 treatment (Supplementary Figure S14C). It is worth noticing that some of the genes identified in this analysis were also found in the previous RNA-seq analysis of combined SBFI-103 and SBFI-1143 treatments of 24 h (Supplementary Figure S9C). The common most upregulated genes are *HMOX1*, *ATF3*, *ILIR1*, Growth differentiation factor 15 (*GDF15*), Anterior Gradient 2 (*AGR2*), *RPLP0P2*, Solute carrier family 7 member 11 (*SLC7A11*), Tribbles pseudokinase 3 (*TRIB3*), and *NIBAN1* and are part of the mechanisms involved in the response to the stress. Simultaneously, *TOP2A*, *RRM2*, *HAS3*, *PHACTR3*, and *COX2* are highly reduced and also identified in the previous analysis (Supplementary Figure S10C). Importantly, Marker of proliferation Ki-67 (*MKI67*), Baculoviral IAP repeat containing 5 (*BIRC5*), MYB proto-oncogene like 2 (*MYBL2*), Anilin (*ANLN*), and Mitochondrially encoded cytochrome c oxidase I and II (*COX1* and *COX2*) genes were amongst the most downregulated markers upon SBFI-1143 treatment at 24 and 48 h (Supplementary Figure S14C).

**Figure 5:**
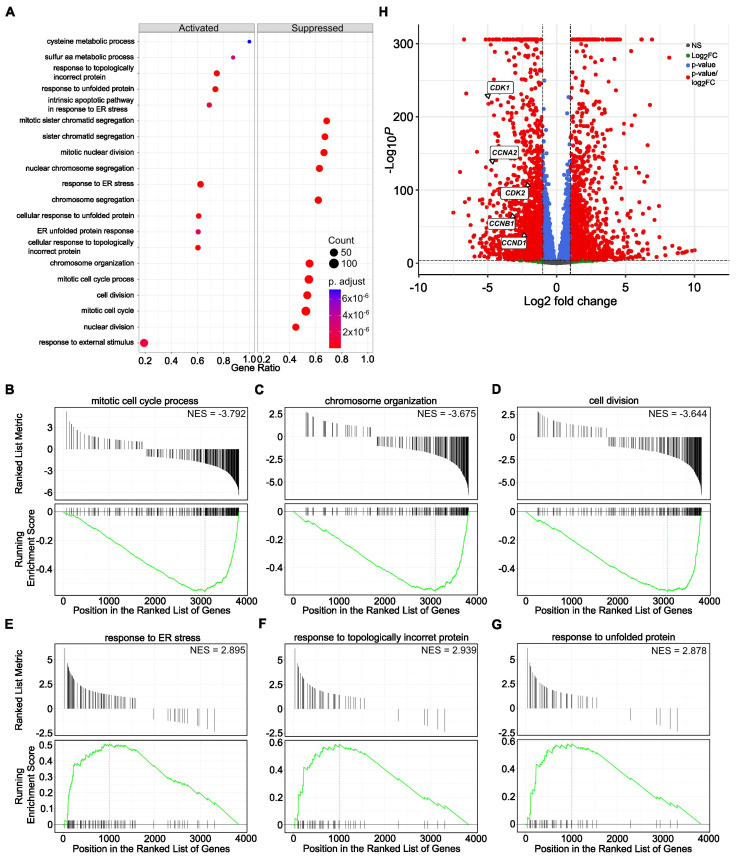
Functional enrichment of activated and suppressed genes and pathways upon SBFI-1143 over two-day treatment in PC-3 PCa cells. (**A**) The top 10 activated and suppressed pathways were identified using gene ontology analysis using a cluster profiler. (**B**–**G**) Gene Set Enrichment Analysis (GSEA) individual gene set enrichment plots of selected three highly downregulated (**B**–**D**) and three highly upregulated (**E**–**G**) pathways in SBFI-1143-treated samples compared to DMSO-treated. NES–normalized enrichment score. (**H**) Enhanced Volcano plot showing five downregulated transcripts of *CDK1*, *CDK2*, *CCNA2*, *CCNB1*, and *CCND1*. Red circles represent genes with |log_2_FC| ≥ 1 and adjusted *p* < 0.05, blue represents genes with adjusted *p* < 0.05 only, and gray represents genes that were neither eligible in conditions of adjusted *p*-value nor |log 2 FC|.

The Gene Ontology and GSEA demonstrated that various processes of cell division, nuclear division, and chromosome and chromatid segregation are among the most suppressed pathways. In contrast, pathways involved in the stress response and response to the topologically incorrect folding of proteins are upregulated (Figure 5A–G). Similarly, the levels of the previously selected five genes, *CDK1*, *CDK2*, *CCNA2*, *CCNB1*, and *CCND1*, involved in the cell cycle progression were significantly decreased in the RNA-seq data (Figure 5H), confirming the shared mechanism of SBFIs’ action.

**Figure 6:**
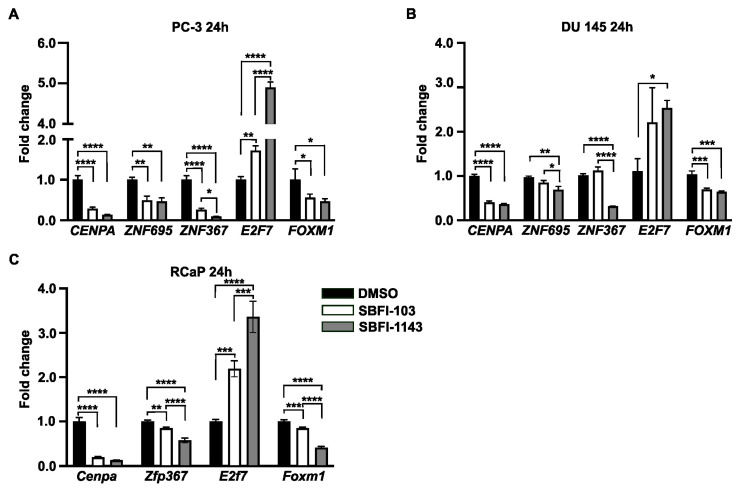
SBFI-1143 affects the expression levels of transcription factors controlling broad spectrum of genes in PCa cells. RT-qPCR analysis of transcription factors after treatment with 15 μmol/L of SBFI-103 or SBFI-1143 for 24 h. (**A**)—PC-3, (**B**)—DU 145, and (**C**)—RcaP. Data are represented as mean ± SD (*N* = 3). One-way ANOVA with Tukey’s multiple range test significance is shown as * *p* < 0.05; ** *p* < 0.001; *** *p* < 0.001; **** *p* < 0.0001.

Current results suggest that SBFIs may have widespread effects on the transcriptomic landscape of prostate cancer cells. Thus, we collated common downregulated and upregulated genes after SBFI-1143 24 and 48 h treatment in PC-3 cells and employed a transcription factor enrichment tool (ChEA3) to identify factors that regulate large sets of these genes. Out of 1215 downregulated genes, 363 genes are regulated by Forkhead box protein M1 (FOXM1), 235 by Centromere Protein A (CENPA), 270 by Zinc finger protein 367 (ZNF367), 354 by E2F Transcription Factor 7 (E2F7), and 259 by the Zinc finger protein 695 (ZNF695) (Supplementary Table S2), and to the same extent, their targets overlap as shown by VennDiagram (Supplementary Figure S15A). It was decided to test whether these transcription factors are also affected in the SBFI-treated PC-3, DU 145, and RCaP cells. RT-qPCR on RNA collected from SBFI-103- and SBFI-1143-treated cells for 24 h was performed. The analysis showed that *CENPA*, *ZNF695*, *ZNF367*, and *FOXM1* transcript levels are significantly reduced upon treatment with SBFIs, while the *E2F7* transcript increased in PC-3 and DU 145 PCa cells (Figure 6A,B). Similarly, *Cenpa*, *Zfp367*, and *Foxm1* genes are downregulated in RCaP cells upon SBFIs treatment, while *E2f7* is upregulated (*Znf695* has not been found) (Figure 6C). The CENPA, ZNF695, ZNF367, and FOXM1 positively regulate cell proliferation, cell cycle progression, and transcriptional activation; E2F7 is known as a transcriptional repressor, whose upregulation may have an additional adverse effect on the transcriptional machinery upon SBFI treatment. Furthermore, we identified common transcription factors of an upregulated set of genes with the following top five shown in the Venn diagram (Supplementary Table S3): Cysteine and serine-rich nuclear protein 1 (*CSRNP1*), Nuclear factor interleukin 3 regulated (*NFIL3*), *DDIT3*, *ATF3*, and *JUN* (Supplementary Figure S15B) and provided their level of expression in RNA-seq datasets in Supplementary Table S1. The listed transcription factors regulate apoptosis, transcriptional repression, and stress response, aligning with RNA-seq analysis results.

**Figure 7:**
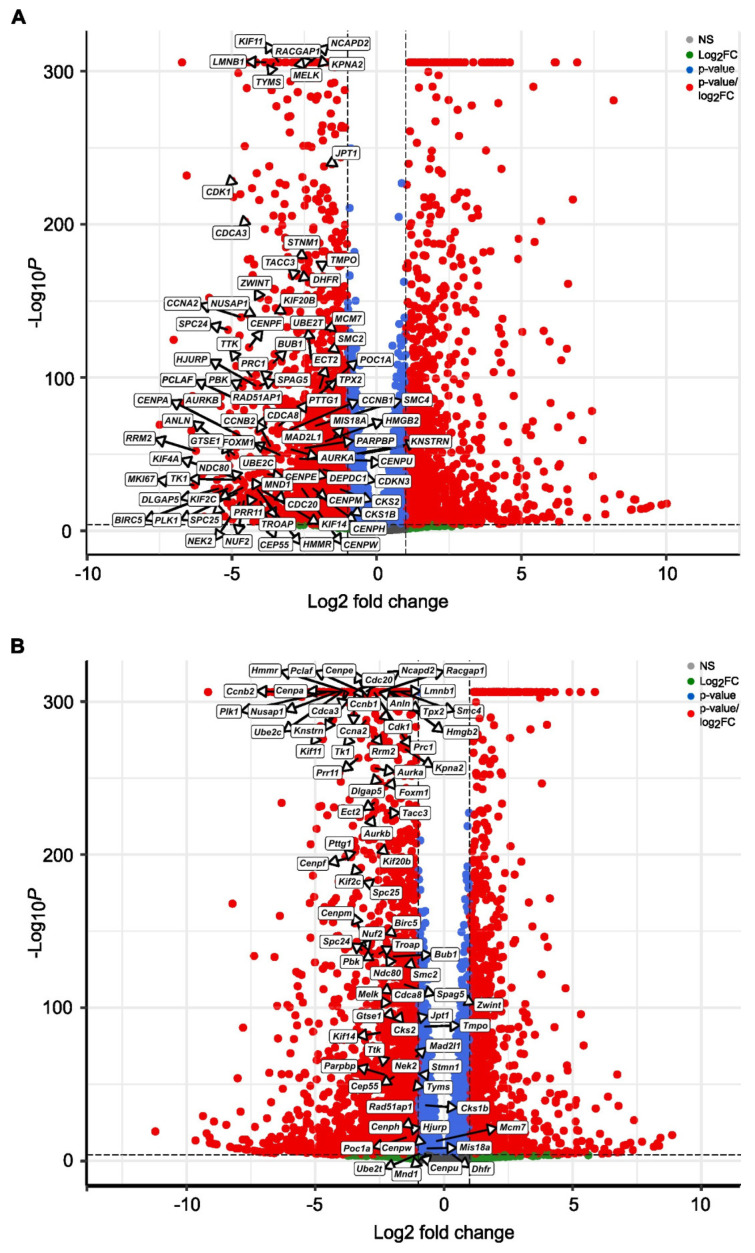
SBFI-1143 regulates lineage plasticity-related prostate cancer genes in PC-3 and RCaP. (**A**) Enhanced Volcano plot showing lineage plasticity-related prostate cancer genes identified in SBFI-1143-treated PC-3 cells compared to controls. (**B**) Enhanced Volcano plot showing lineage plasticity-related prostate cancer genes identified in SBFI-1143-treated RCaP cells compared to controls. Red circles represent genes with |log_2_FC| ≥ 1 and adjusted *p* < 0.05, blue represents genes with adjusted *p* < 0.05 only, and gray represents genes that were neither eligible in conditions of adjusted *p*-value nor |log 2 FC|.

Recent publications identified a set of genes whose increased expression leads to progression from primary to advanced prostate cancer, metastasis, progression toward neuroendocrine phenotype, and development of resistance. Using weighted correlation network analysis, Zhao and colleagues defined lineage plasticity-related gene signature (LPSig) [9]. Initially, we determined that 240 out of 327 genes listed on LPSig were downregulated 24 and 48 h after treatment with SBFI-1143, while six and three were upregulated, respectively, in PC-3 PCa cells (Supplementary Table S4). We assessed whether any of the genes related to the progression toward aggressive adenocarcinoma with a poor prognosis, characterizing the minority population of lineage plasticity-related PC cells (LPCs), are affected by the SBFI-1143 treatment of PC-3 PCa cells [9]. The results demonstrated that 75 and 78 out of 79 genes belonging to the lineage plasticity-related prostate cancer cells were significantly downregulated at 24 and 48 h, respectively, after SBFI-1143 treatment (Figure 7A and Supplementary Table S5). We followed up with results from RNA-seq with SBFI-103’s treatment. Under this condition, only 73 genes out of the lineage plasticity-related prostate cancer cells genes were downregulated, and importantly, their level of downregulation was less noteworthy than that of SBFI-1143 at 24 and 48 h (Supplementary Table S6).

Furthermore, we compared our data to previously identified genes positively correlated with prostate cancer progression toward a castration-independent phenotype [40]. We found that *DNMT1*, *CDK1*, *MKI67*, *CDC20*, *AURKA*, *UBE2C*, *PDGFB*, and *PLK1* were all significantly downregulated in the SBFI-1143-treated PC-3 cells (Supplementary Figure S16A), while *DNTM3A*, *DNMT3B*, *EED*, *EZH2*, and *CD24* were showing a trend of decreasing (Supplementary Figure S16A and Supplementary Table S7). These genes were found in the set of genes inhibited by SBFI-103; however, their level of inhibition was lower. Noteworthy, SBFI-1143 treatment led to significant downregulation of *GATA2* and *HOXB13*. The former acts as a chromatin remodeler at enhancer sites to regulate the expression and activity of AR in CRPC, while the latter is the chromatin remodeling factor and, in addition, interacts with AR and binds to AR’s target loci [41,42,43,44]. In addition, SBFI-1143 decreases the expression of *BRCA1*, *BRCA2*, and *PARP1*, inhibiting positive regulatory feedback for the AR pathway [45,46,47,48]. Of these, only *GATA2*, *BRCA1*, and *BRCA2* were significantly reduced by SBFI-103 (Supplementary Table S7). Likewise, treatment of RCaP cells with SBFI-1143 within 24 h resulted in significant downregulation of genes driving a malignant, castration-independent phenotype version of PCa, remodeling chromatin, and providing a positive feedback loop for the AR pathway (Figure 7B, Supplementary Figure S16B and Supplementary Table S7).

In summary, the data show that SBFI-103 and SBFI-1143 negatively regulate PCa cell proliferation, block cell cycle progression, and induce apoptosis, with the latter having a more pronounced effect. The comprehensive RNA-seq analysis showed that most downregulated genes belong to the various pathways controlling cell division, while upregulated genes are primarily involved in stress response. In addition, we showed that SBFI-1143 has a more widespread effect than SBFI-103 by modifying the levels of multiple transcription factors and genes involved in the progression of primary PCa toward metastasis.

## 4. Discussion

The current study analyzed the impact of three SBFI compounds on the viability, proliferation, cell cycle, and apoptosis of the PC-3 PCa cell line. We demonstrated that SBFI-1143, a member of the truxillic-acid monoester (TAME) family of third-generation FABP5 inhibitors, has a potent suppressive effect on PCa growth compared to the first- and second-generation inhibitors (SBFI-26 and SBFI-103, respectively). These results aligned with recent data showing that SBFI-1143 potently inhibits PCa growth and induces apoptosis after 72 h of treatment. Importantly, we showed that the strong inhibitory effect of SBFI-1143 on proliferation was observed in the three-dimensional system in three PCa cells (PC-3, DU 145, and RCaP) that more accurately reproduces in vivo conditions. At the same time, SBFI-103 has a diminished capacity for reducing PCa viability under these conditions compared to SBFI-1143.

Comprehensive transcriptomic analysis demonstrated that both SBFI-103 and SBFI-1143 suppressed the activity of signaling pathways related to cell cycle, cell division, chromatin, and chromosome segregation and activated pathways regulating response to ER stress, misfolded proteins, and apoptosis, with SBFI-1143 showing higher potency. These results aligned impeccably with the presented results of the impacts of these two compounds on cell cycle, proliferation, and apoptosis. Importantly, RT-qPCR and Western blot analyses confirmed that five selected cell cycle-related genes identified by RNA-seq were significantly reduced in three tested PCa cells. The levels of reductions in CDK1, CDK2, Cyclins A2, B1, and D1 were markedly higher upon SBFI-1143 treatment than SBFI-103. The results demonstrate uniform response of metastatic and castration-resistant prostate cancer cell lines to both compounds.

As RNA-seq analysis consistently revealed that most inhibited pathways are related to cell division, we wanted to establish whether the same transcription factors control downregulated genes. The ChEA3 analysis identified several transcription factors regulating the bulk of these genes. The top five selected were assessed by RT-qPCR, and it showed that four of them, *CENPA*, *FOXM1*, *ZNF367*, and *ZNF695*, were significantly downregulated, while *E2F7* upregulated upon SBFI-1143 treatment in PC-3 and DU 145. Similar results were obtained in RCaP cells. *CENPA*, *FOXM1*, *ZNF367*, and *ZNF695* are overexpressed in PCa and positively correlate with PCa proliferation [49,50,51,52]. E2F7 is a transcriptional repressor and negatively regulates the expression of many genes involved in cell cycle progression. It has been shown to play the role of tumor suppressor and oncogene in a context-dependent manner [53]. However, its role in PCa is poorly understood, showing contradictory data regarding its expression level during tumor development [54,55].

The increased availability of patients’ biospecimens from various stages of PCa disease from benign, primary, metastatic, and neuroendocrine, and next-generation sequencing (transcriptomics and proteomics) combined with integrative analysis allowed for the identification of the roadmap of transcriptomic changes during tumor progression. RNA-seq analysis showed that several genes, *DNMT1*, *CDK1*, *MKI67*, *CDC20*, *AURKA*, *UBE2C*, *PDGFB*, and *PLK1*, previously positively correlated with the progression of PCa, are markedly downregulated upon SBFI-103 and SBFI-1143 treatment [40]. Importantly, the inhibition of these genes is significantly higher in SBFI-1143-treated compared to SBFI-103-treated PC-3 cells. The products of these genes are overexpressed in PCa compared to normal tissue and regulate chromosome segregation, cell proliferation, stemness, epithelial-to-mesenchymal transition, cancer stem cell formation, and resistance to chemotherapy. Interestingly, the study demonstrated that SBFI-1143 has a potent inhibitory effect on lineage plasticity-related genes (LPSig) identified by Zhao and colleagues [9]. Their integrative analysis identified 327 genes altered during transdifferentiating toward aggressive PCa. The majority of these genes belong to the pathways regulating the cell cycle, cell division, DNA recombination and repair, signal transduction, and transcription. Furthermore, we showed that SBFI-1143 inhibits levels of genes that are identified in the lineage of plasticity-related prostate cancer cells and characterized by low AR expression, stemness characteristics, high levels of Hyaluronan Mediated Motility Receptor (HMMR), and are associated with a poor prognosis. The majority of the 79 signature genes were downregulated by SBFI-1143 treatment in PC-3 and RCaP cells, demonstrating the ability of the tested compound to efficiently modify the transcriptomic landscape of PCa and decrease the levels of genes that are correlated with the malignant PCa phenotype.

Previous studies examining the role of SBFI compounds on the transcriptome in cancer progression were conducted in breast cancer models. The authors used SBFI-26 at a higher concentration (100 μM) and observed changes in pathways involved in metabolism, genetic information processing, cellular processes, and transcription [56]. Specifically, they showed that SBFI-26 under these conditions induces ferroptosis. In the presented data, the ferroptosis pathway was not identified as one of the most induced. However, genes such as *ATF3*, *ATF4*, *HMOX1*, *SAT1*, and *CHAC1* were upregulated in our dataset, demonstrating similar responses to SBFI compounds. It is crucial to notice that *ATF3*, *HMOX1*, and *CHAC1* were identified as the top twenty DEG and were classified as stress-response genes. Another study showed that SBFI-26 regulates the expression of *FOXM1* and WNT signaling in ovarian granulosa cells [57]. Similarly, in the analysis, *FOXM1* was decreased upon SBFI-1143 in three tested PCa cell lines. In addition, several genes involved in regulating the WNT pathway or components of this pathway, such as *Sfrp2*, *Aspn*, *Rspo2*, *Srpx*, *Cd248*, *Tmem119*, *Ptn*, *Nid2*, *Fzd4*, *Lrp4/5*, *Wnt4*, and *Wnt10b*, were downregulated in RCaP cells upon SBFI-1143. In contrast, *SRPX*, *PTN*, *FZD4*, *LRP4/5*, *LGR5*, *WNT4*, *WNT10B*, and *WNT7A/7B* were downregulated in PC-3 cells. The data indicate that the third-generation SBFI-1143 compound has a considerably more substantial effect on the activity of various signaling pathways, such as WNT, TGFβ, and MAPK, regulating proliferation, viability, migration, and metastasis, thereby impacting the expression levels of a plethora of genes leading to cell death. As such, further investigation of the impact of SBFI-1143 on multiple PCa cells and in vivo models with a focus on pathway intersection will enhance understanding of the mechanism of action of SBFI compounds on PCa development, progression, and metastasis.

## 5. Conclusions

These studies are the first to characterize SBFI compounds’ impact on the PCa transcriptomic landscape. It was demonstrated that targeting FABP5 with SBFI-1143 or SBFI-103 leads to substantial remodeling of the transcriptomic PCa landscape by downregulating multiple genes involved in the cell cycle and division and significantly reduces the expression levels of the signature genes involved in the progression towards aggressive PCa. Further studies employing an in vivo model and combinatorial treatment with other drugs, such as taxanes [29], provide a promising path to therapy of primary and malignant PCa.

## 6. Patents

Iwao Ojima and Martin Kaczocha report that the National Institutes of Health and Artero Biosciences provided financial support. They have patents issued to the Research Foundation of the State University of New York. Other authors declare no competing financial interest.

## Data Availability

Total RNA-sequencing data generated and discussed in this study have been deposited in NCBI’s Gene Expression Omnibus (GEO) (RRID: SCR_005012) and are accessible through the GEO Series accession numbers: GSE297940, GSE297941, and GSE309891. The Supplementary Figure, Supplementary Figure Legends, and Supplementary Tables are available using the following link: https://www.mdpi.com/article/10.3390/cancers17233723/s1. All other data generated in this study are available upon request from the corresponding author.

## Supplementary Materials

The following supporting information can be downloaded at: https://www.mdpi.com/article/10.3390/cancers17233723/s1. Supplementary Figure S1. Treatment with SBFIs inhibited the proliferation of PC-3 cells in a dose and time-dependent manner. Supplementary Figure S2. SBFI-1143-treatment significantly inhibited cell cycle progression in PC-3 cells after 24 h of treatment compared to SBFI-26 and SBFI-103. Supplementary Figure S3. SBFI-1143-treatment significantly inhibited cell cycle progression in PC-3 cells after 48 h of treatment compared to SBFI-26 and SBFI-103. Supplementary Figure S4. SBFI-1143 treatment significantly inhibited cell cycle progression in PC-3 cells after 72 h of treatment compared to SBFI-26 and SBFI-103. Supplementary Figure S5. Representative results of FACS analysis of cell cycle performed using PC-3 cells stained with PI. Supplementary Figure S6. SBFI-1143 significantly induces apoptosis in PC-3 cells compared to SBFI-26 and SBFI-103 with 24 h treatment. Supplementary Figure S7. SBFI-1143 significantly induces apoptosis in PC-3 cells compared to SBFI-26 and SBFI-103 within 48 h of treatment. Supplementary Figure S8. SBFI-1143 significantly induces apoptosis in PC-3 cells compared to SBFI-26 and SBFI-103 with 72 h of treatment. Supplementary Figure S9. Representative results of FACS analysis of PC-3 cells stain with AnnexinV/PI apoptosis kit. Supplementary Figure S10. Principal component analysis (PCA) and heatmaps of PC-3 cells treated with SBFI-103 and SBFI-1143. Supplementary Figure S11. Principal component analysis (PCA) and heatmaps of RCaP cells treated with SBFI-103 and SBFI-1143. Supplementary Figure S12. Functional enrichment of activated and suppressed genes and pathways upon SBFI-103 and SBFI-1143 treatment of RCaP cells. Supplementary Figure S13. Commonly activated and suppressed pathways upon SBFI-103 and SBFI-1143 treatment of PC-3 and RCaP cells. Supplementary Figure S14. Principal component analysis (PCA) and heatmaps of PC-3 cells treated with SBFI-1143 over two days. Supplementary Figure S15. Top 5 transcription factors regulating downregulated and upregulated transcripts in SBFI-1143-treated PC-3 cells. Supplementary Figure S16. Enhanced Volcano plots demonstrate that the levels of genes positively correlate with prostate cancer progression towards castration-independent phenotype and AR regulation are downregulated in SBFI-1143-treated PC-3 and RCaP cells. Supplementary Table S1. The list of genes regulating cell cycle progression and transcription factors regulating commonly downregulated and upregulated genes by SBFI-103 and SBFI-1143 in PC-3 and RCaP. Supplementary Table S2. The list of transcription factors regulating commonly downregulated genes by SBFI-1143 in PC-3 cells at 24 and 48 h. Supplementary Table S3. The list of transcription factors regulating commonly upregulated genes by SBFI-1143 in PC-3 cells at 24 and 48 h. Supplementary Table S4. The list of genes belonging to the LPSig identified at 24 and 48 h of treatment with SBFI-1143. Supplementary Table S5. The list of genes belonging to the LPC signature identified at 24 and 48 h of treatment with SBFI-1143. Supplementary Table S6. The list of genes belonging to the LPC signature identified at 24 h of treatment with SBFI-103. Supplementary Table S7. The list of genes driving the development and progression of PCa identified during SBFI-103 and SBFI-1143 treatments.

## Abbreviations

The following abbreviations are used in this manuscript:

ACTB	Actin
ADGFR1	Adhesion G protein-coupled receptor F
AGR2	Anterior gradient 2
AGTR2	Angiotensin II Receptor Type 2
ANLN	Anillin
AR	Androgen Receptor
ASPN	Asporin
ATF3	Activating Transcription Factor 3
ATF4	Activating Transcription Factor 4
BIRC5	Baculoviral IAP repeat-containing protein 5 or Survivin
BRCA1	Breast cancer susceptibility gene 1
BRCA2	Breast cancer susceptibility gene 2
CCNA2	Cyclin A2
CCNB1	Cyclin B1
CCND1	Cyclin D1
CDC20	Cell division cycle protein 20 homolog
CDK1	Cyclin-dependent kinase 1
CDK2	Cyclin-dependent kinase 2
CENPA	Centromere protein A
CD24	CD24 antigen
CD248	Endosialin
CHAC1	ChaC Glutathione Specific Gamma-Glutamylcyclotransferase 1
ChEA3	ChIP-X Enrichment Analysis 3
COL1A1	Collagen type I alpha 1 chain
COL3A1	Collagen type III alpha 1 chain
COX1	Cyclooxygenase-1
COX2	Cyclooxygenase-2
CRABP1	Cellular retinoic acid binding protein 1
CRPC	Castration-resistant prostate cancer
CSRNP1	Cysteine and serine rich nuclear protein 1
DDIT4	DNA Damage-Inducible Transcript 4
DMSO	Dimethyl sulfoxide
DNMT1	DNA methyltransferase 1
DNMT3A	DNA methyltransferase 3A
DNTM3B	DNA methyltransferase 3B
DPT	Dermatopontin
EED	Embryonic ectoderm development
EFEMP1	Epidermal Growth Factor-Containing Fibulin-Like Extracellular Matrix Protein 1
EZH2	Enhancer of Zeste Homolog 2
E2F7	E2F Transcription Factor 7
FABP5	Fatty Acid-Binding Protein 5
FAM111B	FAM111 Trypsin Like Peptidase B
FOXM1	(Forkhead box protein M1)
FZD4	Frizzled-4
GAS1	Growth arrest-specific protein 1
GAS6	Growth arrest-specific protein 6
GATA2	GATA binding protein 2
GDF15	Growth Differentiation Factor 15
GSEA	Gene set enrichment analysis
HAS3	Hyaluronan Synthase 3
HMMR	Hyaluronan-mediated motility receptor
HMOX1	Heme oxygenase 1
HOXB13	Homeobox B13
HRP	Horseradish peroxidase
IGFBP5	Insulin-like Growth Factor-Binding Protein 5
IL1R1	Interleukin 1 receptor, type I
JUN	Transcription factor Jun
LGR5	Leucine-rich repeat containing G protein-coupled receptor 5
LPC	A subpopulation of prostate cancer cells with a role in trans-differentiation, recurrence and poor prognosis
LPSig	Lineage plasticity-related signature
LRP4	Low-density lipoprotein receptor-related protein 4
LRP5	Low-density lipoprotein receptor-related protein 5
MAPK	Mitogen-activated protein kinase
MKI67	Marker of Proliferation Ki-67
μmol/L	Micromoles per liter
Mmol/L	Millimoles per liter
mPC	Metastatic prostate cancer
MYBL2	Myeloblastosis oncogene-like 2
MYC	C-MYC proto-oncogene
NFIL3	Nuclear Factor Interleukin 3 Regulated
NIBAN1	Niban Apoptosis Regulator 1
NID2	Nidogen-2
PARP1	Poly(ADP-Ribose) Polymerase 1
PCa	Prostate cancer
PDGFB	Platelet derived growth factor subunit B
PHACTR3	Phosphatase and actin regulator 3
PLK1	Polo-like kinase 1
PTN	Pleiotrophin
RIN	RNA integrity number
RNA-seq	RNA-sequencing
RN7SK	7SK small nuclear RNA
RPLP0P2	Ribosomal Protein Lateral Stalk Subunit P0 Pseudogene 2
RRM2	Ribonucleotide Reductase Regulatory Subunit M2
RSPO	R-spondin-1
SAT1	Spermidine/spermine N1-acetyltransferase 1
SBFI	Stony Brook FABP5 inhibitor
SESN2	Sestrin-2
SFRP2	Secreted frizzled-related protein 2
SLC7A11	Solute carrier family 7 member 11
SRPX	Sushi repeat containing protein X-linked
S100a4	S100 calcium-binding protein A4
TAME	Truxillic-acid monoester
TGFβ	Transforming growth factor beta
TMEM119	Transmembrane protein 119
TOP2A	DNA topoisomerase II alpha
TRIB3	Tribbles Homolog 3
Tris-HCl	Tris(hydroxymethyl)aminomethane hydrochloride
UBE2C	Ubiquitin-conjugating enzyme E2C
WNT4	Wnt Family Member 4
WNT7A	Wnt Family Member 7A
WNT7B	Wnt Family Member 7B
WNT10B	Wnt Family Member 10B
ZNF367	Zinc Finger Protein 367
ZNF695	Zinc Finger Protein 695
