# Supplementary material for: SBFI Inhibitors Reprogram Transcriptomic Landscape of Prostate Cancer Cells Leading to Cell Death
Source: Cancers (Basel). 2025 Nov 21;17(23):3723. doi: 10.3390/cancers17233723 (PMC12691007; doi:10.3390/cancers17233723)
Supplement: Supplementary file 1 [file cancers-17-03723-s001.zip › SUPPLEMENTARY FIGURE AND TABLE LEGEND.pdf]

SUPPLEMENTARY FIGURE LEGEND

Supplementary Figure S1. Treatment with SBFIs inhibited the proliferation of PC-3 cells in a dose and time-dependent manner. PC-3 cells were treated with various concentrations of compounds: (**A**) 5 µmol/L, (**B**) 10 µmol/L, (**C**) 20 µmol/L, (**D**) 50 µmol/L, and (**E**) 100 µmol/L of SBFI-26, 103, or 1143 for 24, 48, and 72 hours. The measurement of the control (0.2% DMSO) was defined as 100%. Cells were counted using a cell counter at each time point, and data represents mean ±SD (*N=3*).

Supplementary Figure S2. SBFI-1143-treatment significantly inhibited cell cycle progression in PC-3 cells after 24 h of treatment compared to SBFI-26 and SBFI-103. Cell cycle progression was assessed by combining PI stain and flow cytometry. Bar graphs show the effect of different concentrations of (**A**) 5 µmol/L, (**B**) 10 µmol/L, (**C**) 20 µmol/L, (**D**) 50 µmol/L, and (**E**) 100 µmol/L of SBFIs. Data represents mean ±SD with N=3. Two-way ANOVA with Tukey's multiple range test significance is shown as *p<0.05; **p<0.01; ***p<0.001; ****p<0.0001.

Supplementary Figure S3. SBFI-1143-treatment significantly inhibited cell cycle progression in PC-3 cells after 48 h of treatment compared to SBFI-26 and SBFI-103. Cell cycle progression was assessed by combining PI stain and flow cytometry. Bar graphs show the effect of different concentrations of (**A**) 5 µmol/L, (**B**) 10 µmol/L, (**C**) 20 µmol/L, (**D**) 50 µmol/L, and (**E**) 100 µmol/L of SBFIs. Data represents mean ±SD with N=3. Two-way ANOVA with Tukey's multiple range test significance is shown as *p<0.05; **p<0.01; ***p<0.001; ****p<0.0001.

Supplementary Figure S4. SBFI-1143-treatment significantly inhibited cell cycle progression in PC-3 cells after 72 h of treatment compared to SBFI-26 and SBFI-103. Cell cycle progression was assessed by combining PI stain and flow cytometry. Bar graphs show the effect of different concentrations of (**A**) 5 µmol/L, (**B**) 10 µmol/L, (**C**) 20 µmol/L, (**D**) 50 µmol/L, and (**E**) 100 µmol/L of SBFIs. Data represents mean ±SD with N=3. Two-way ANOVA with Tukey's multiple range test significance is shown as *p<0.05; **p<0.01; ***p<0.001; ****p<0.0001.

Supplementary Figure S5. Representative results of FACS analysis of cell cycle performed using PC-3 cells stained with PI. Cell cycle progression was assessed by combining PI stain and flow cytometry. The images are shown as representative results after 24h (**A**), 48h (**B**), and 72h (**C**) of treatment with DMSO and 15 µmol/L of SBFIs.

Supplementary Figure S6. SBFI-1143 significantly induces apoptosis in PC-3 cells compared to SBFI-26 and SBFI-103 with 24-hour treatment. PC-3 cells were treated with various concentrations of SBFI-26, SBFI-103, SBFI-1143, and DMSO as control and collected

at 24 hours. The level of apoptosis was assessed using Annexin V/PI stain with flow cytometry. Bar graphs show the effect of different concentrations of (**A**) 5 µmol/L, (**B**) 10 µmol/L, (**C**) 20 µmol/L, (**D**) 50 µmol/L, and (**E**) 100 µmol/L of SBFI compounds. Data are represented as mean ±SD (N=3). Two-way ANOVA with Tukey's multiple range test significance is shown as *p<0.05; **p<0.01; ***p<0.001; ****p<0.0001.

Supplementary Figure S7. SBFI-1143 significantly induces apoptosis in PC-3 cells compared to SBFI-26 and SBFI-103 within 48 hours of treatment. PC-3 cells were treated with various concentrations of SBFI-26, SBFI-103, SBFI-1143, and DMSO as control and collected at 48 hours. The level of apoptosis was assessed using Annexin V/PI stain with flow cytometry. Bar graphs show the effect of different concentrations of (**A**) 5 µmol/L, (**B**) 10 µmol/L, (**C**) 20 µmol/L, (**D**) 50 µmol/L, and (**E**) 100 µmol/L of SBFIs. Data are represented as mean ±SD (N=3). Two-way ANOVA with Tukey's multiple range test significance is shown as *p<0.05; **p<0.01; ***p<0.001; ****p<0.0001.

Supplementary Figure S8. SBFI-1143 significantly induces apoptosis in PC-3 cells compared to SBFI-26 and SBFI-103 with 72 hours of treatment. PC-3 cells were treated with various concentrations of SBFI-26, SBFI-103, SBFI-1143, and DMSO as control and collected at 72 hours. The level of apoptosis was assessed using Annexin V/PI stain with flow cytometry. Bar graphs show the effect of different concentrations of (**A**) 5 µmol/L, (**B**) 10 µmol/L, (**C**) 20 µmol/L, (**D**) 50 µmol/L, and (**E**) 100 µmol/L of SBFIs. Data are represented as mean ±SD (N=3). Two-way ANOVA with Tukey's multiple range test significance is shown as *p<0.05; **p<0.01; ***p<0.001; ****p<0.0001.

Supplementary Figure S9. Representative results of FACS analysis of PC-3 cells stain with AnnexinV/PI apoptosis kit. The level of apoptosis was assessed using Annexin V/PI stain with flow cytometry. The images are shown as representative results after 24h (**A**), 48h (**B**), and 72h (**C**) of treatment with DMSO and 15 µmol/L of SBFIs.

Supplementary Figure S10. Principal component analysis (PCA) and heatmaps of PC-3 cells treated with SBFI-103 and SBFI-1143. RNA sequencing analysis was conducted on PC-3 cells treated with vehicle control (0.2% DMSO) or SBFI-103 or SBFI-1143 (15 µmol/L) for 24 hours. (**A**) Principal component analysis (PCA) RNA-seq shows the primary separation of samples based on the treatment. (**B**) Proximity comparison presented as a sample-to-sample heatmap confirms a strong correlation with respect to biological replicates and differences between treatment conditions. (**C**) The heatmap represents the hierarchical clustering of genes and samples and shows the top twenty differentially expressed genes between tested conditions.

Supplementary Figure S11. Principal component analysis (PCA) and heatmaps of RCaP cells treated with SBFI-103 and SBFI-1143. RNA sequencing analysis was conducted on RCaP cells treated with vehicle control (0.2% DMSO) or SBFI-103 or SBFI-1143 (15 µmol/L)

for 24 hours. (**A**) Principal component analysis (PCA) RNA-seq shows the primary separation of samples based on the treatment. (**B**) Proximity comparison presented as a sample-to-sample heatmap confirms a strong correlation with respect to biological replicates and differences between treatment conditions. (**C**) The heatmap represents the hierarchical clustering of genes and samples and shows the top twenty differentially expressed genes between tested conditions.

Supplementary Figure S12. Functional enrichment of activated and suppressed genes and pathways upon SBFI-103 and SBFI-1143 treatment of RCaP cells. (**A**) The top 10 activated and suppressed pathways were identified using gene ontology analysis using a cluster profiler. (**B-G**) Gene Set Enrichment Analysis (GSEA) individual gene set enrichment plots of selected three highly downregulated (**B-D**) and three highly upregulated (**E-G**) pathways in SBFI-treated samples compared to DMSO-treated. NES–normalized enrichment score. (**H**) Enhanced Volcano plot showing five downregulated transcripts of *Cdk1*, *Cdk2*, *Ccna2*, *Ccnb1*, and *Ccnd1*. Red circles represent genes with $|\log_2 FC| \geq 1$ and adjusted $p < 0.05$, blue represents genes with adjusted $p < 0.05$ only, and grey represents genes that were neither eligible in conditions of adjusted p-value nor |log 2 FC|.

Supplementary Figure S13. Commonly activated and suppressed pathways upon SBFI-103 and SBFI-1143 treatment of PC-3 and RCaP cells. (**A**) Activated and (**B**) suppressed pathways in RNA-seq data sets of PC-3 and RCaP cells. Data analysis performed with set of genes with $|\log_2 FC| \geq 1$ and adjusted $p < 0.05$.

Supplementary Figure S14. Principal component analysis (PCA) and heatmaps of PC-3 cells treated with SBFI-1143 over two days. RNA sequencing analysis was conducted on PC-3 cells treated with vehicle control (DMSO) or SBFI-1143 (15 µmol/L) for 24 and 48 hours. (**A**) Principal component analysis (PCA) RNA-seq shows the primary separation of samples based on the treatment. (**B**) A proximity comparison presented as a sample-to-sample heatmap confirms a strong correlation with respect to biological replicates and differences between treatment conditions. (**C**) The heatmap represents the hierarchical clustering of genes and samples and shows the top twenty differentially expressed genes between tested conditions.

Supplementary Figure S15. Top 5 transcription factors regulating downregulated and upregulated transcripts in SBFI-1143-treated PC-3 cells. (**A**) The genes downregulated by treatment with SBFI-1143 for 24 and 48 hours were inputted in the ChEA3 browser. The diagram represents the top 5 transcription factors with the number of regulated genes shown above each bar. (**B**) The genes upregulated by treatment with SBFI-1143 for 24 and 48 hours were inputted in the ChEA3 browser. The diagram represents the top 5 transcription factors with the number of regulated genes shown above each bar.

Supplementary Figure S16. **Enhanced Volcano demonstrates that the levels of genes positively correlate with prostate cancer progression towards castration-independent phenotype and AR regulation are downregulated in SBFI-1143-treated PC-3 and RCaP cells.** (**A**) Enhanced Volcano plot showing genes driving the progression of PCa and AR regulation identified in SBFI-1143-treated PC-3 compared to control. Red circles represent genes with $|log2FC| \geq 1$ and adjusted $p < 0.05$, blue represents genes with adjusted $p < 0.05$ only, and grey represents genes that were neither eligible in conditions of adjusted p-value nor |log 2 FC|. (**B**) Enhanced Volcano plot showing genes driving the progression of PCa and AR regulation identified in SBFI-1143-treated RCaP compared to control. Red circles represent genes with $|log2FC| \geq 1$ and adjusted $p < 0.05$, blue represents genes with adjusted $p < 0.05$ only, and grey represents genes that were neither eligible in conditions of adjusted p-value nor |log 2 FC|.

Supplementary Table S1. The list of genes regulating cell cycle progression and transcription factors regulating commonly downregulated and upregulated genes by SBFI-103 and SBFI-1143 in PC-3 and RCaP. Clear boxes represent genes with $|log_2FC| \geq 1$ and adjusted $p < 0.05$, grey boxes represent genes with adjusted $p <0.05$ only, pink boxes genes with $|log_2FC| \geq 1$ only, and blue boxes represent genes that were neither eligible in conditions of adjusted p-value nor |log 2 FC|.

Supplementary Table S2. The list of transcription factors regulating commonly downregulated genes by SBFI-1143 in PC-3 cells at 24 and 48 hours.

Supplementary Table S3. The list of transcription factors regulating commonly upregulated genes by SBFI-1143 in PC-3 cells at 24 and 48 hours.

Supplementary Table S4. The list of genes belonging to the LPSig identified at 24 and 48 hours of treatment with SBFI-1143.

Supplementary Table S5. The list of genes belonging to the LPC signature identified at 24 and 48 hours of treatment with SBFI-1143.

Supplementary Table S6. The list of genes belonging to the LPC signature identified at 24 hours of treatment with SBFI-103.

Supplementary Table S7. The list of genes driving the development and progression of PCa identified during SBFI-103 and SBFI-1143 treatments. Clear boxes represent genes with $|log_2FC| \geq 1$ and adjusted $p < 0.05$, grey boxes represent genes with adjusted $p <0.05$ only, and blue boxes represent genes that were neither eligible in conditions of adjusted p-value nor |log 2 FC|.
