# Supplementary material for: SBFI Inhibitors Reprogram Transcriptomic Landscape of Prostate Cancer Cells Leading to Cell Death
Source: Cancers (Basel). 2025 Nov 21;17(23):3723. doi: 10.3390/cancers17233723 (PMC12691007; doi:10.3390/cancers17233723)
Supplement: Supplementary file 1 [file cancers-17-03723-s001.zip › Supplementary Figures.pdf]

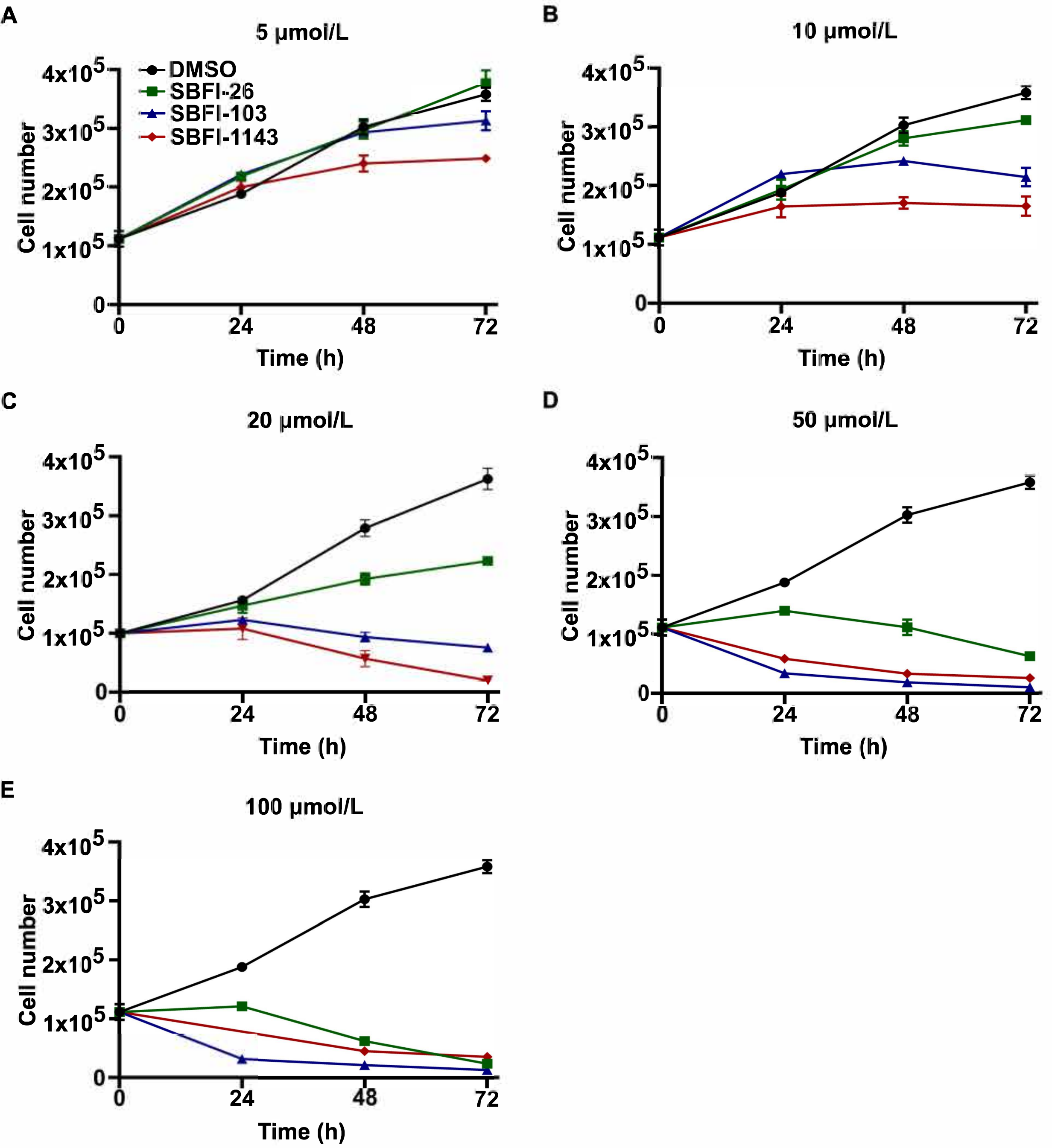

Supplementary Figure S1.

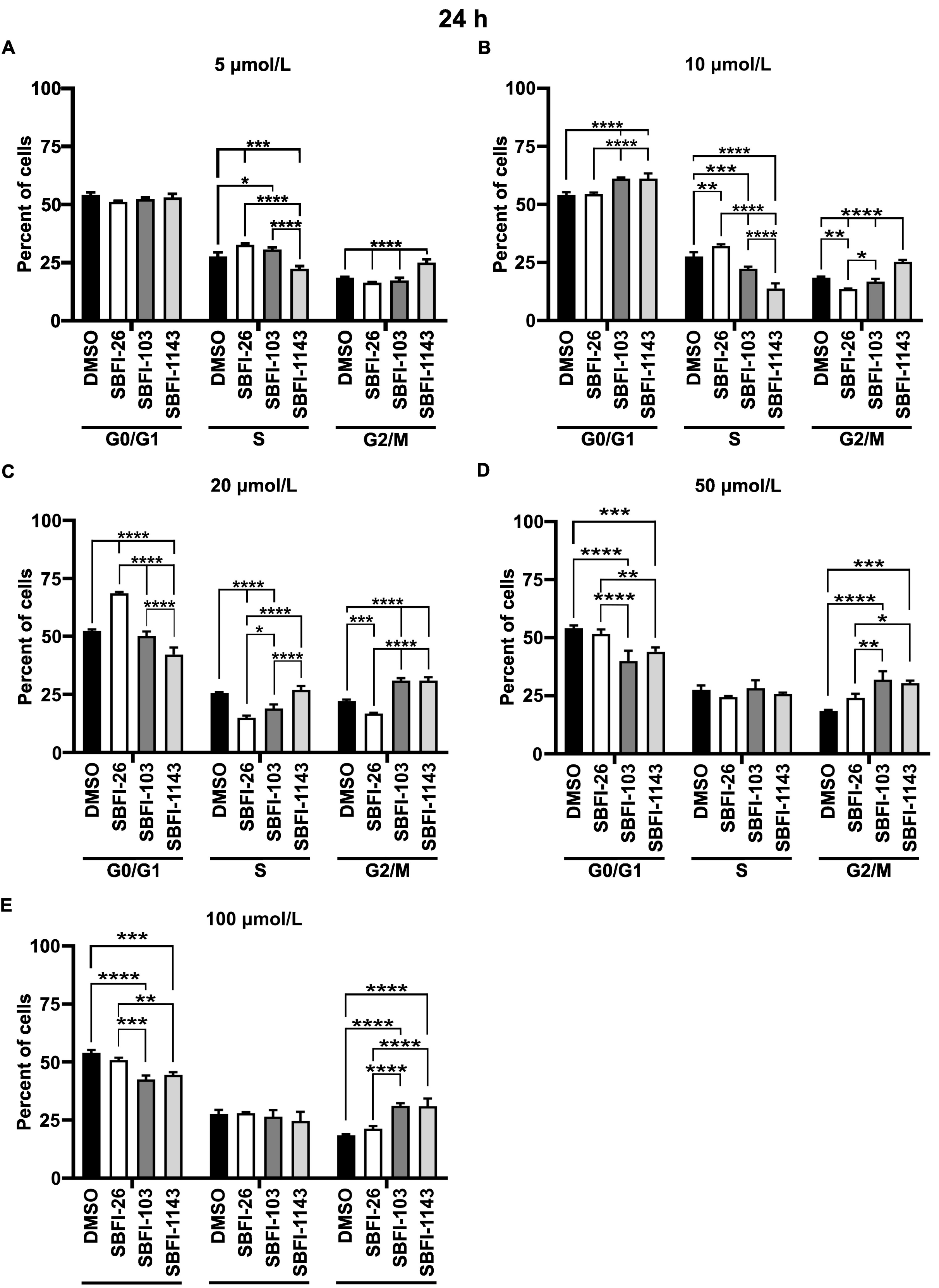

Supplementary Figure S2.

48 h

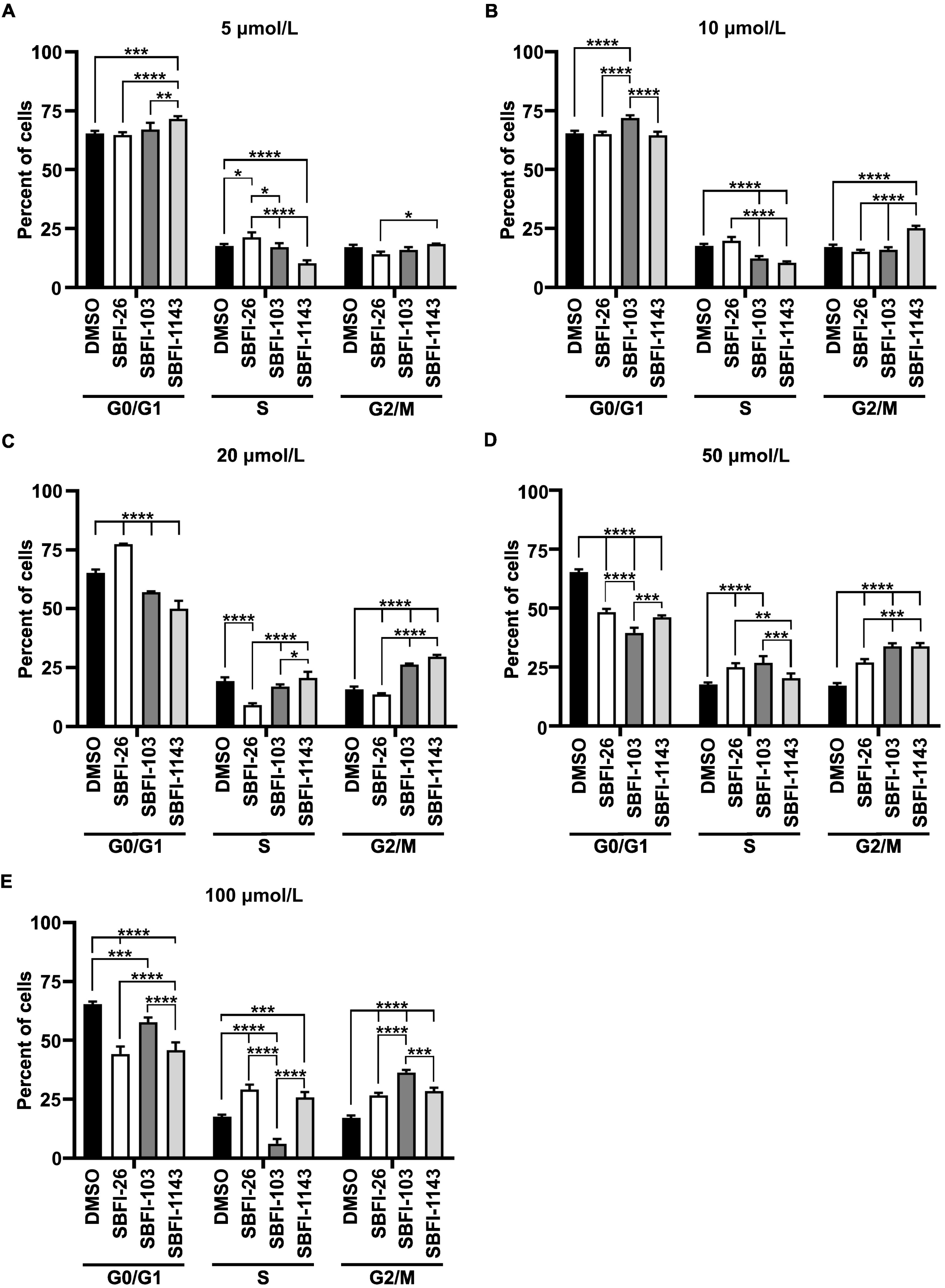

Supplementary Figure S3.

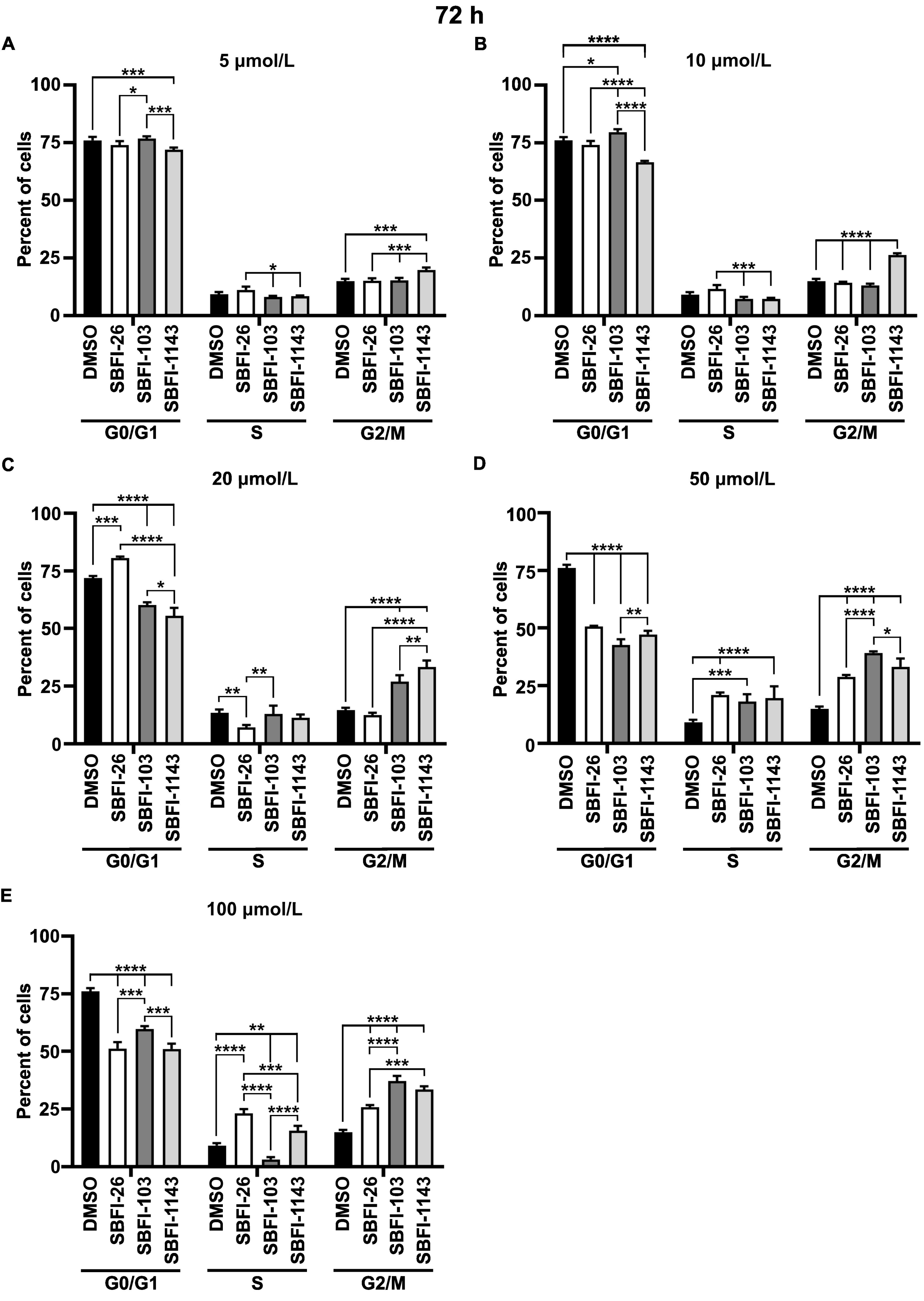

Supplementary Figure S4.

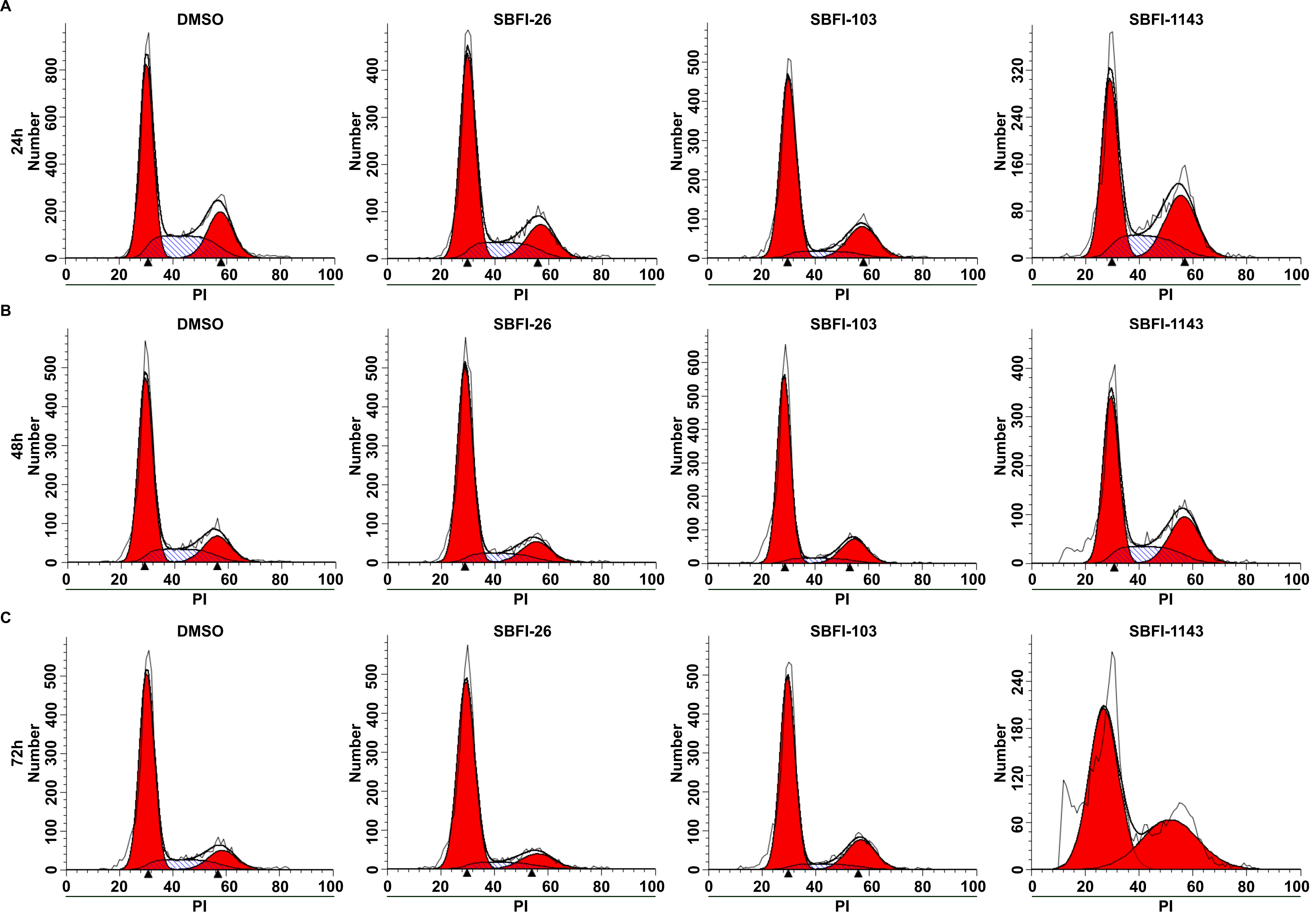

Supplementary Figure S5.

48 h

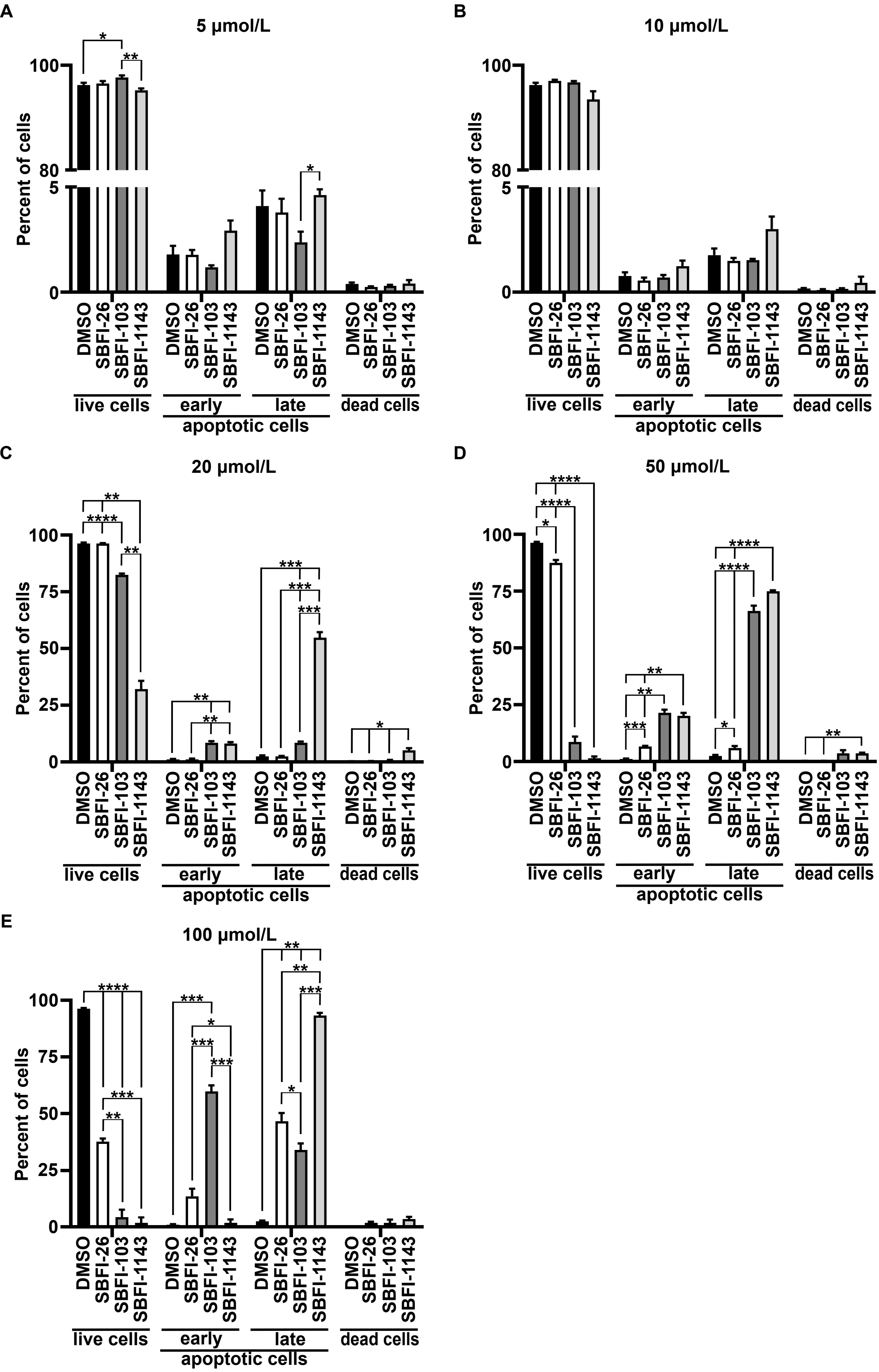

Supplementary Figure S7.

72 h

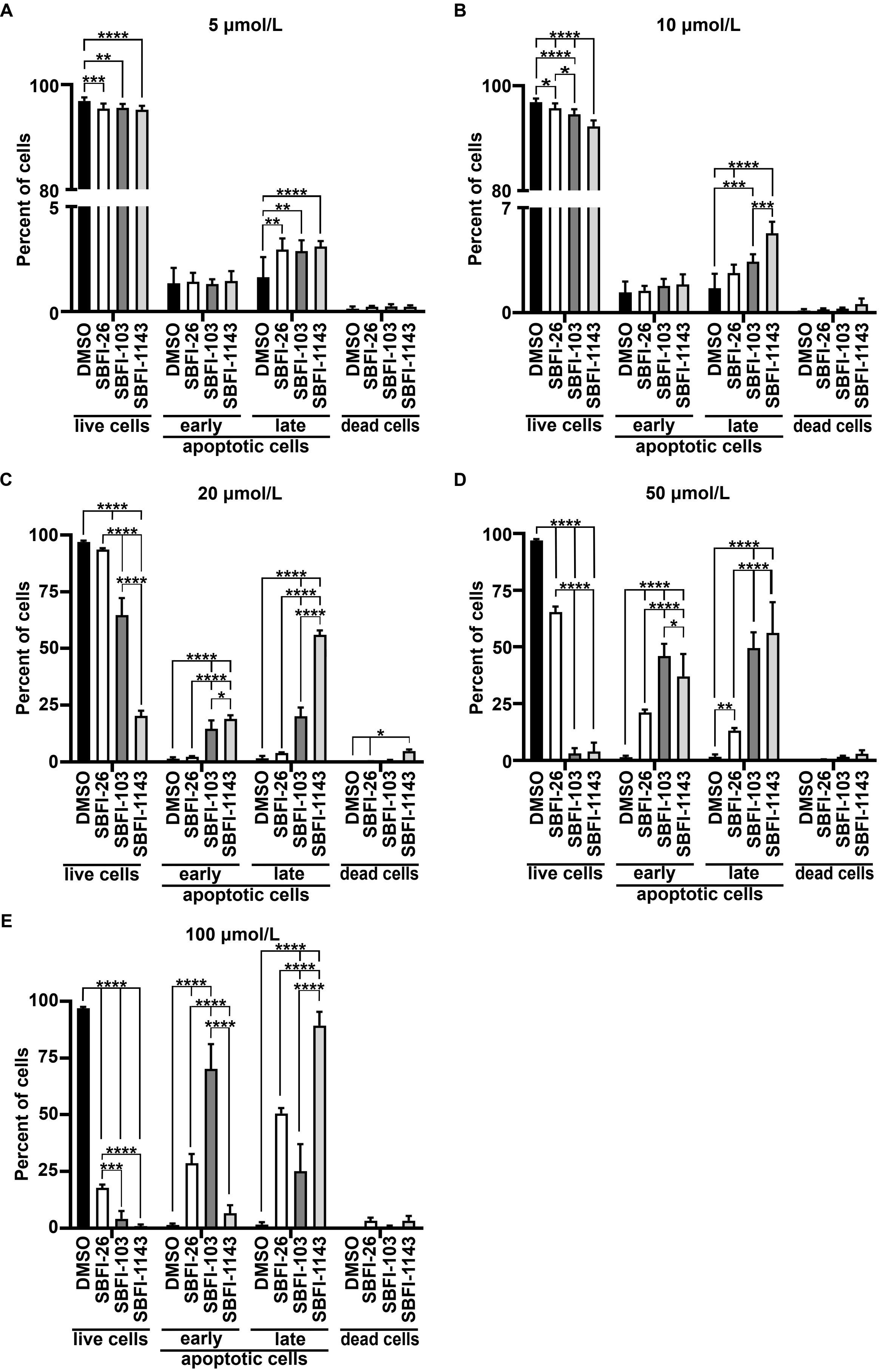

Supplementary Figure S8.

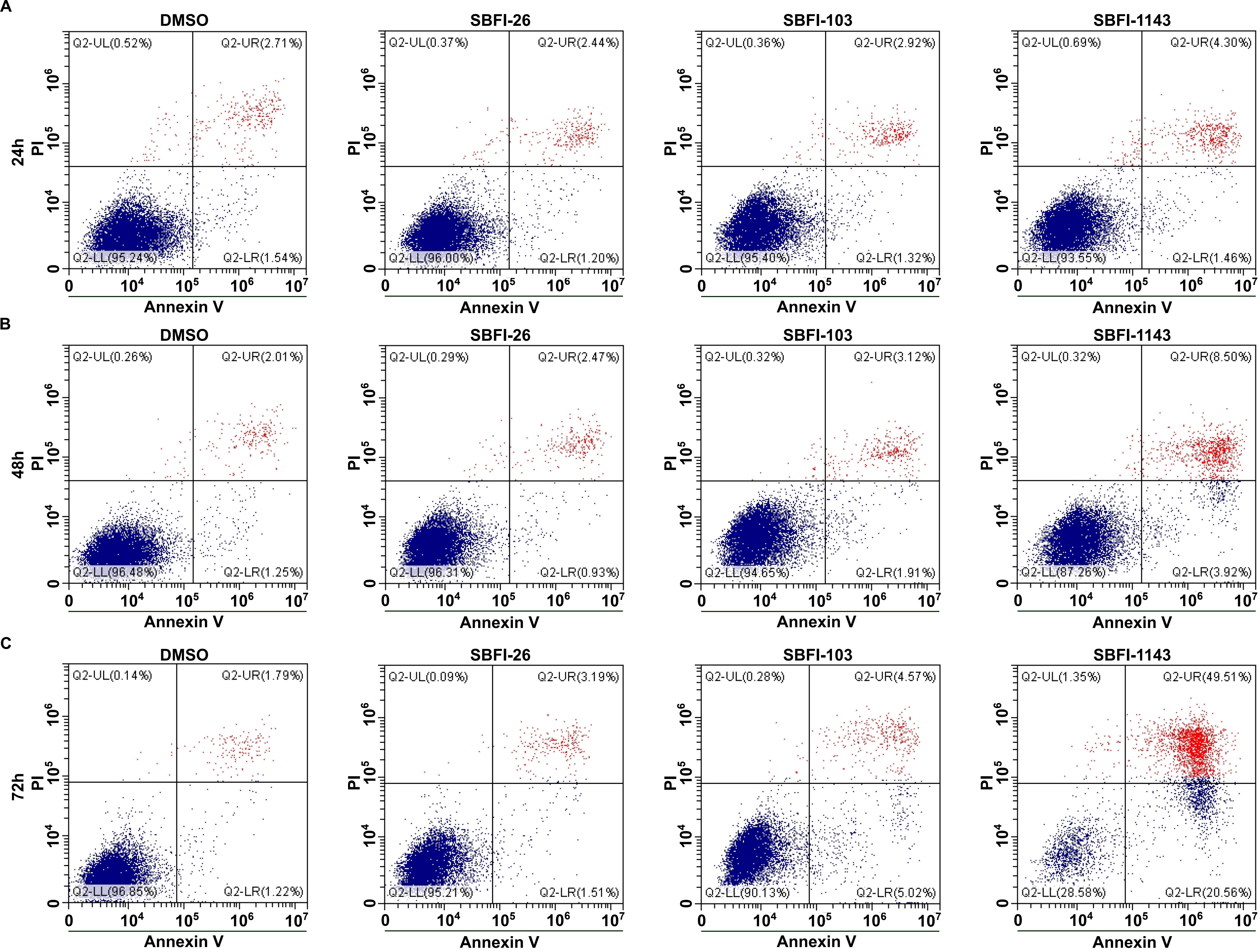

Supplementary Figure S9.

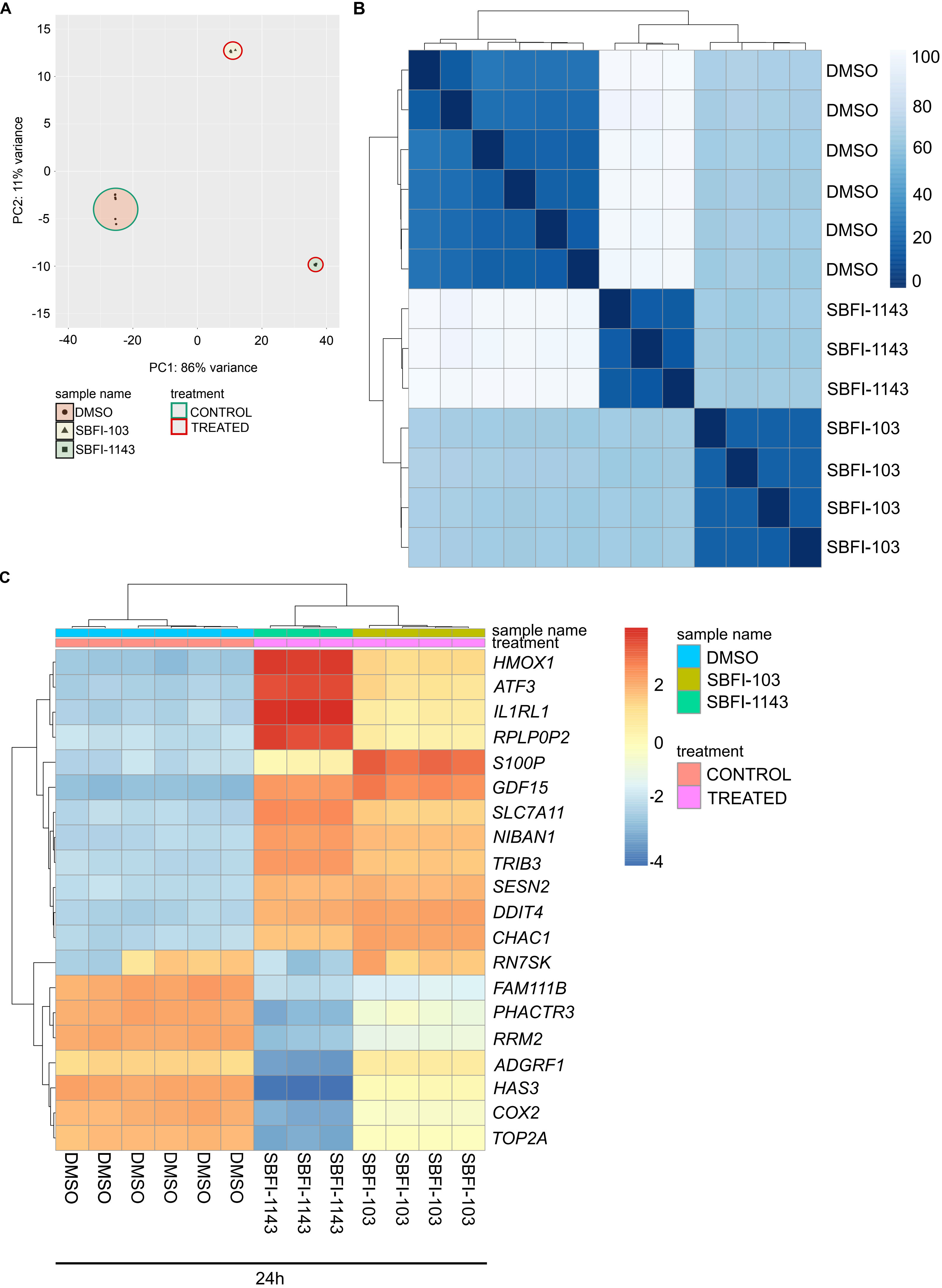

Supplementary Figure S10.

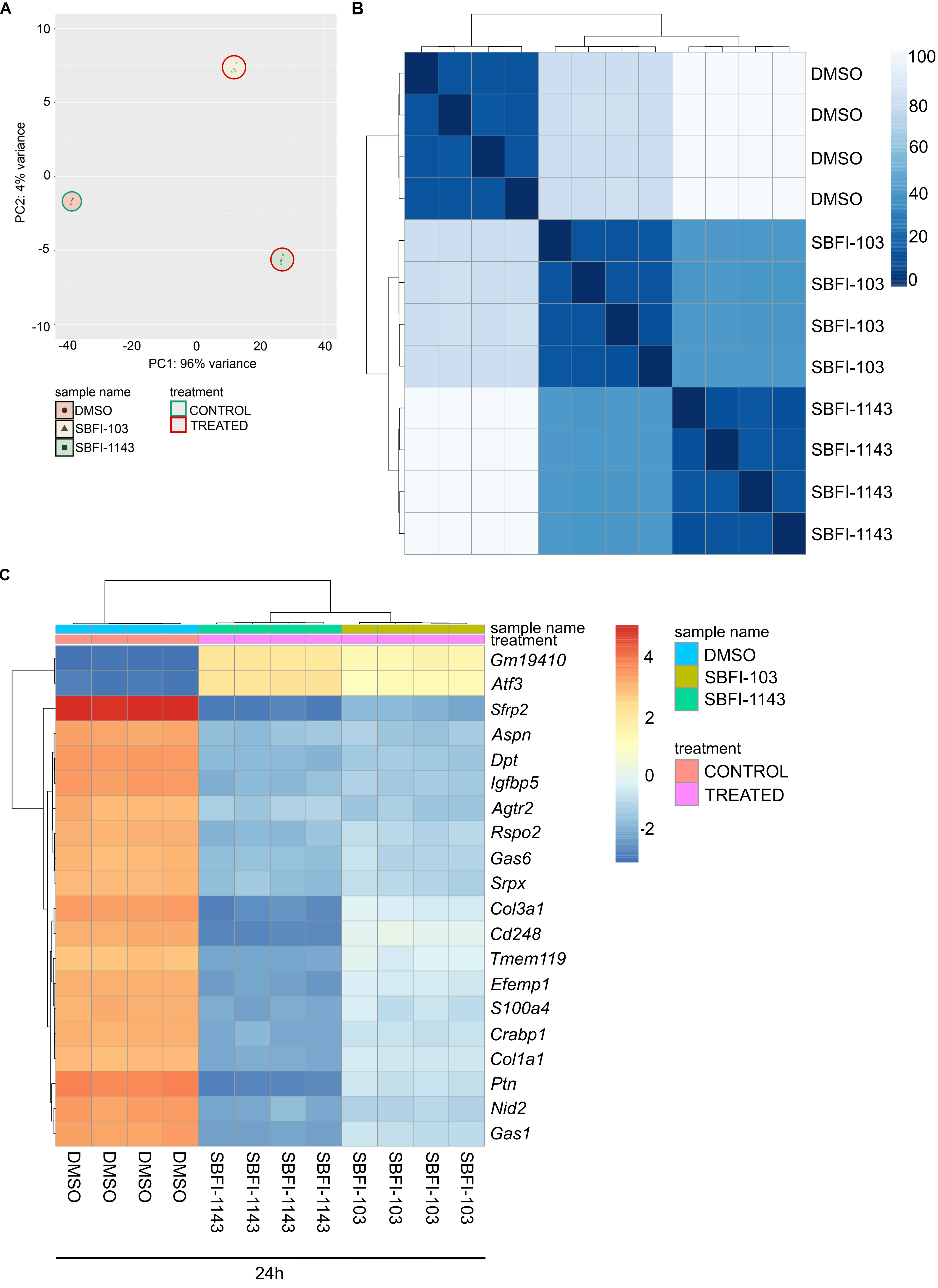

Supplementary Figure S11.

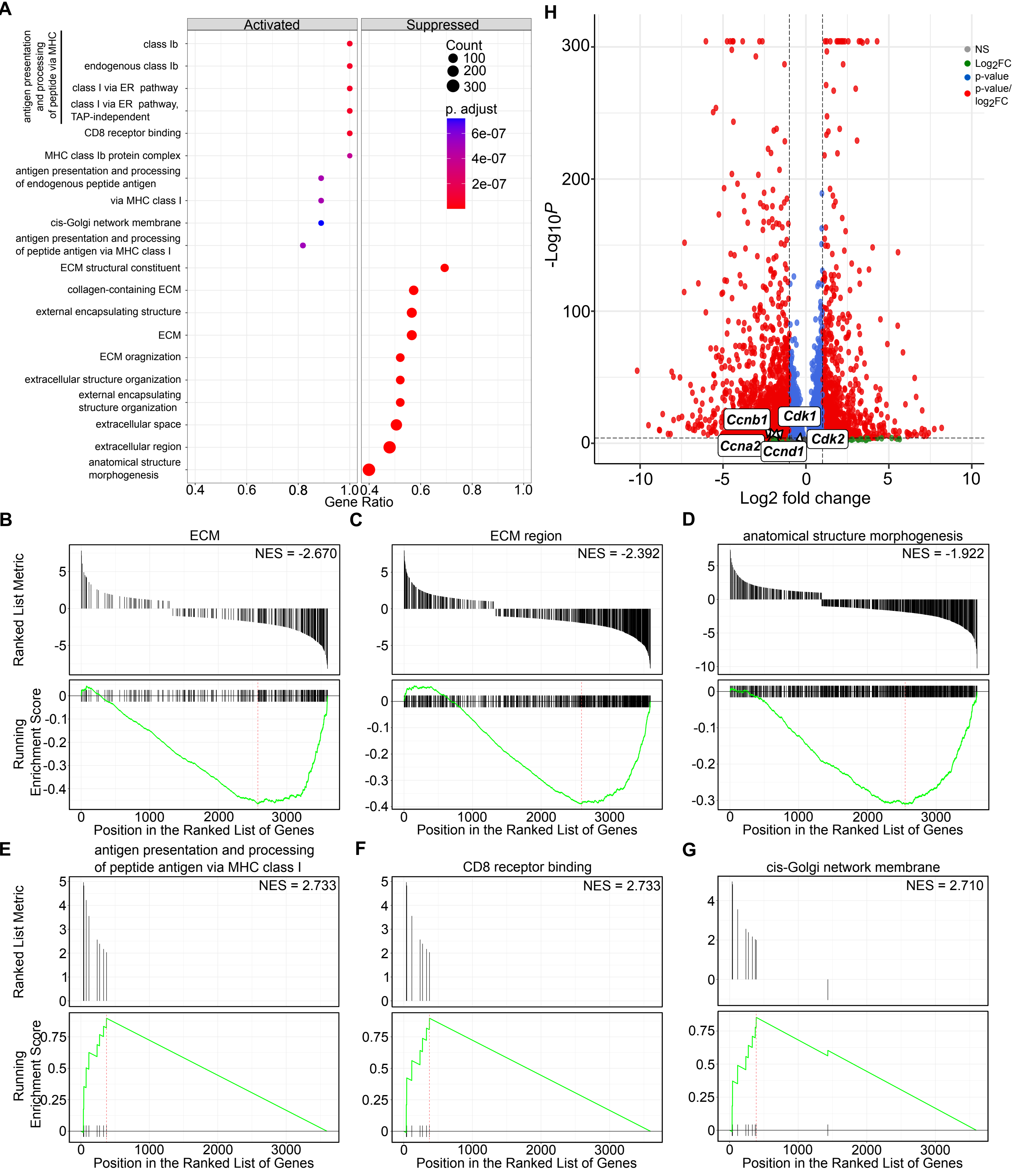

Supplementary Figure S12.

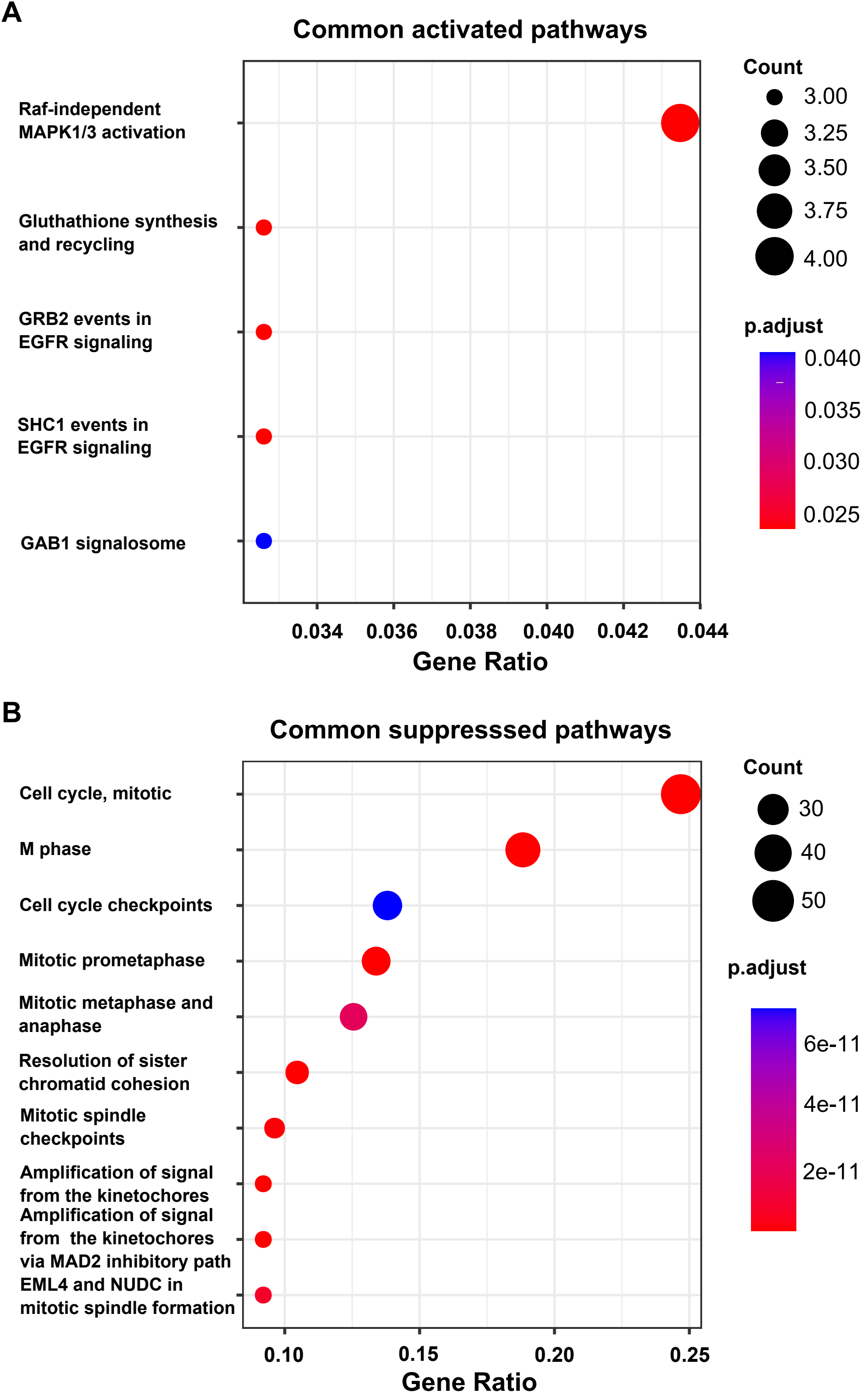

Supplementary Figure S13.

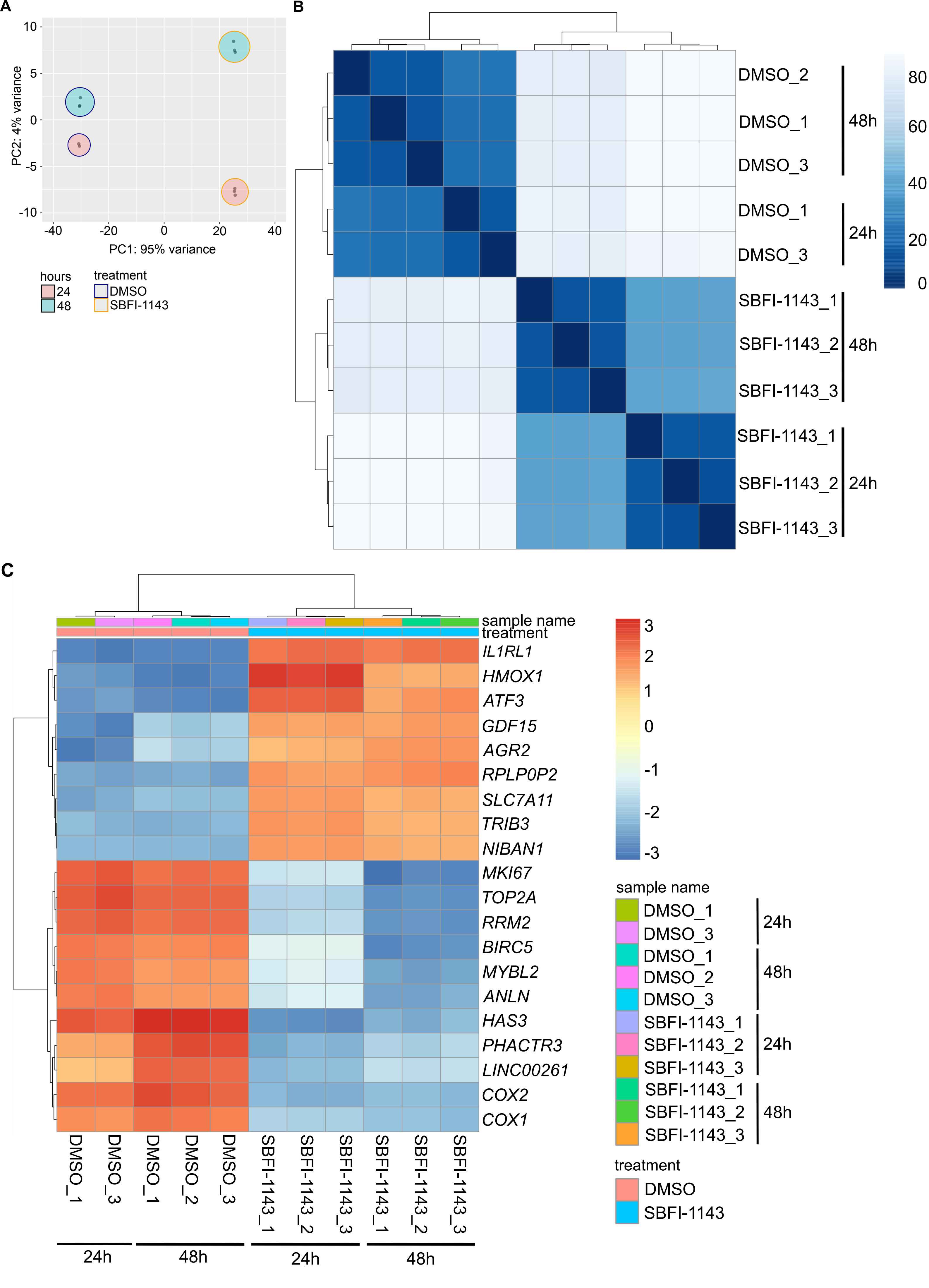

Supplementary Figure S14.

A

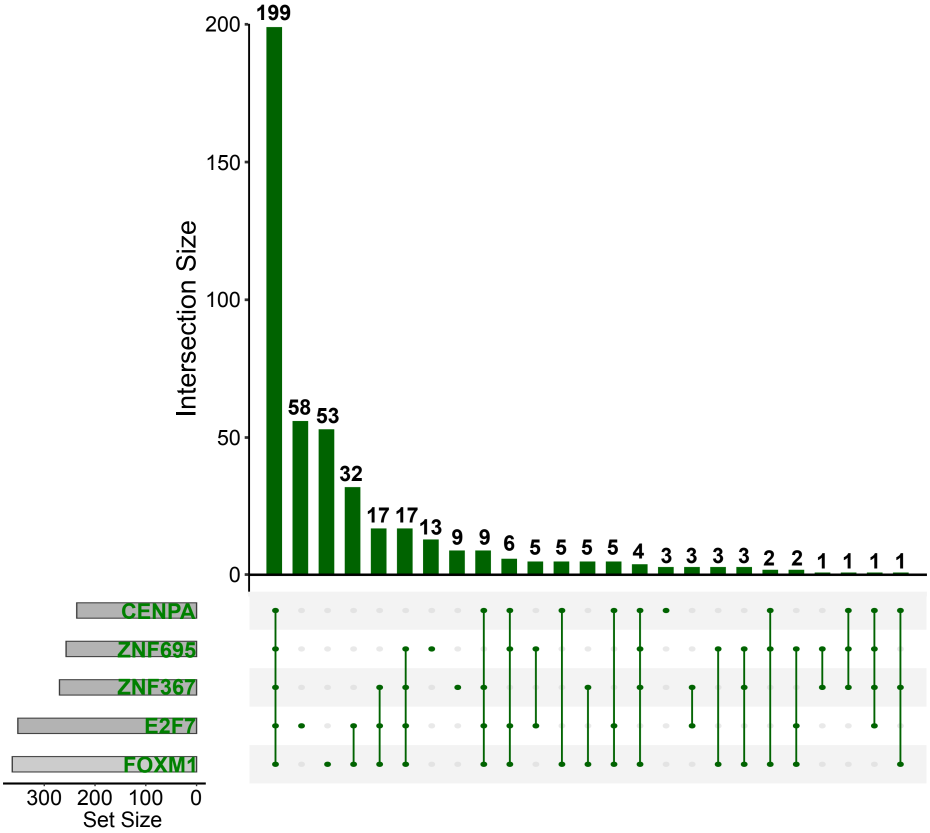

B

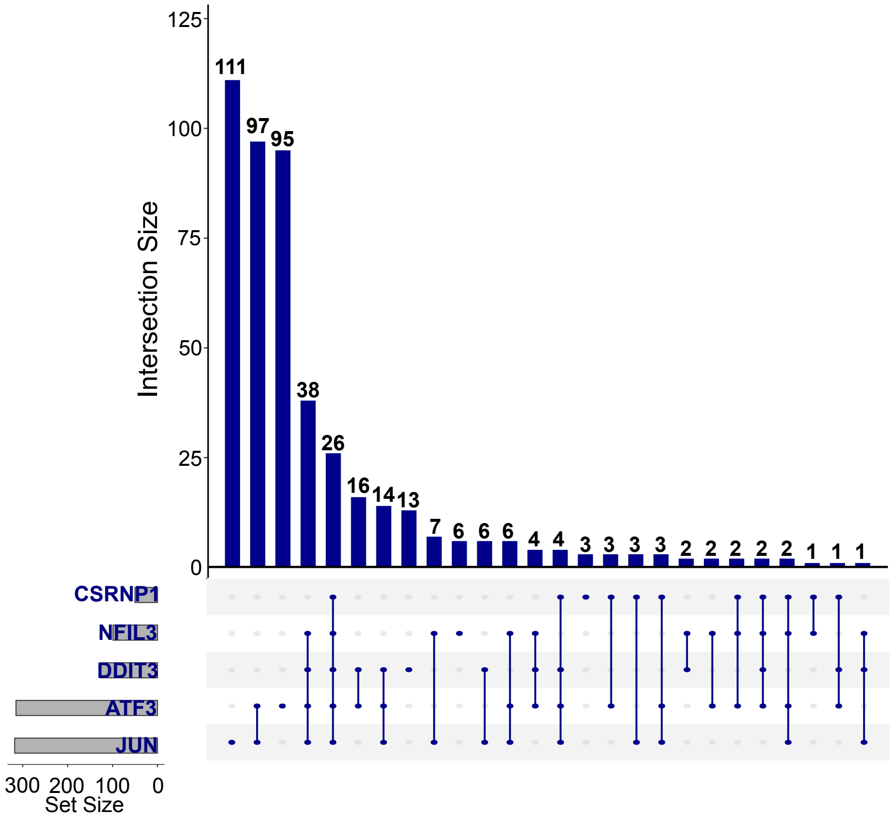

Supplementary Figure S15.

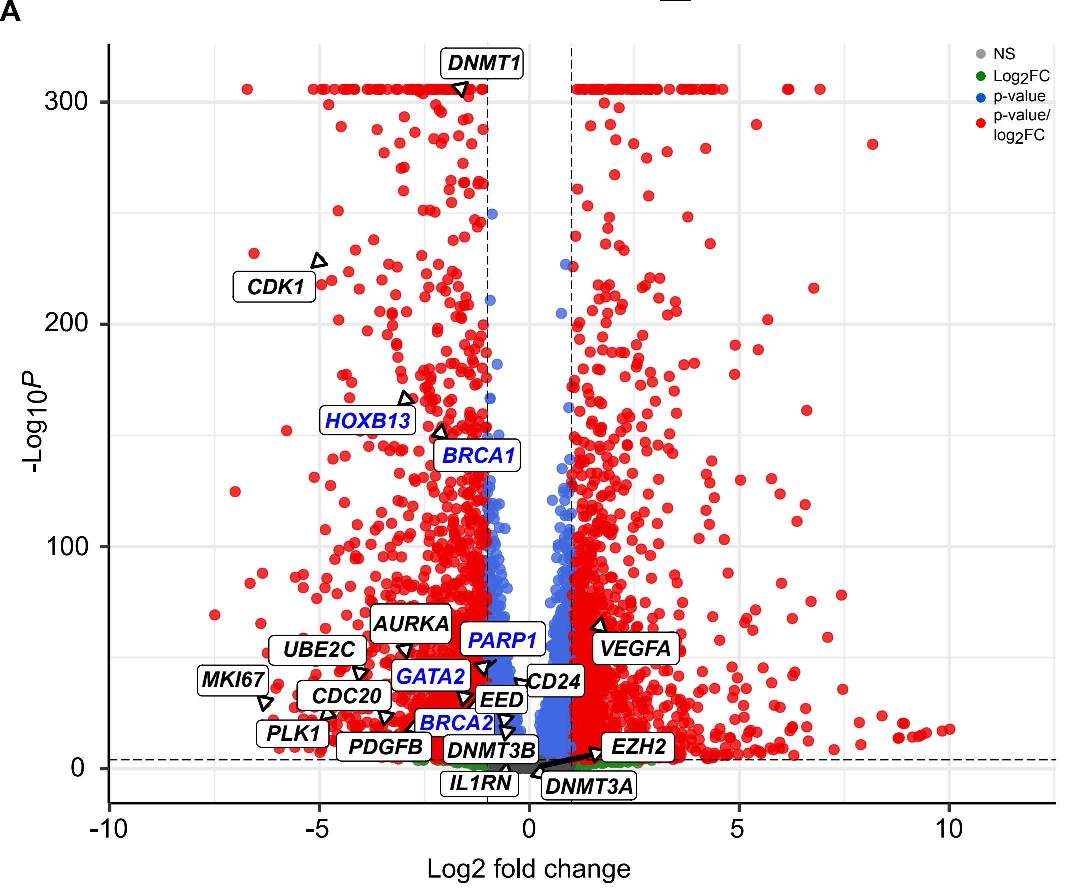

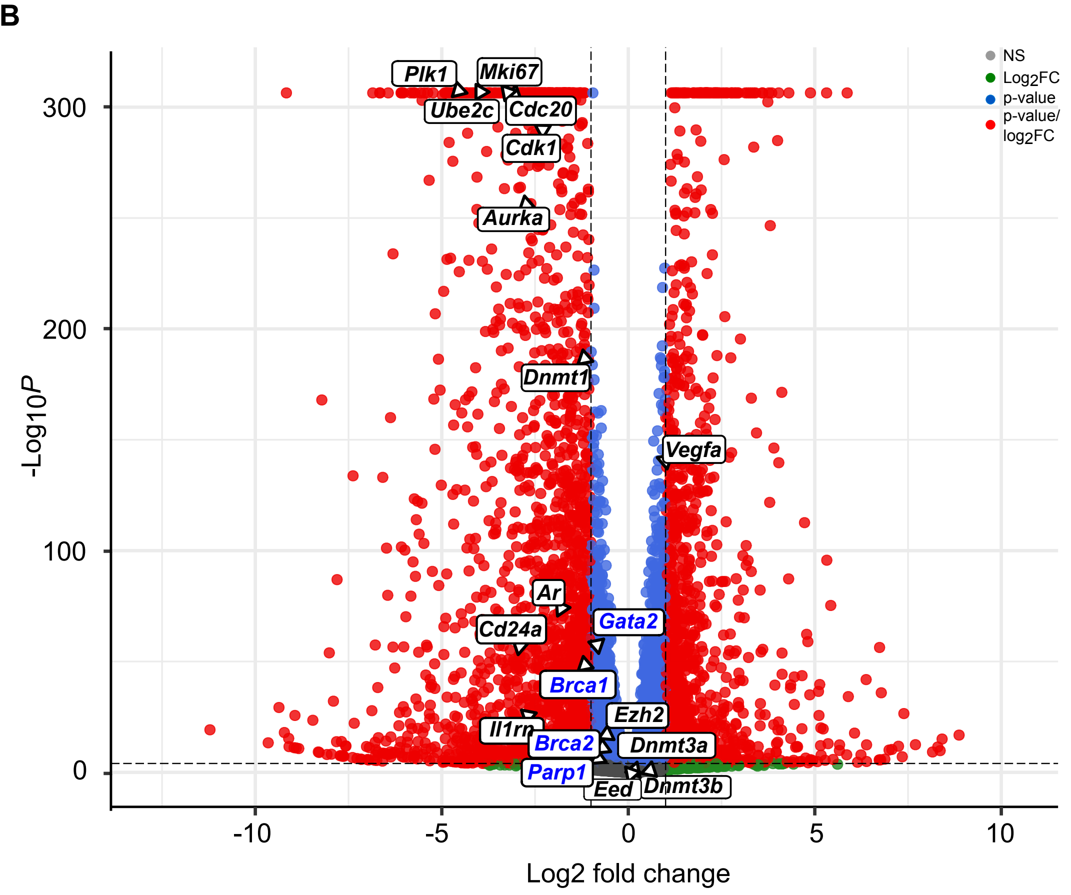

Supplementary Figure S16.
