# Supplementary material for: SBFI Inhibitors Reprogram Transcriptomic Landscape of Prostate Cancer Cells Leading to Cell Death
Source: Cancers (Basel). 2025 Nov 21;17(23):3723. doi: 10.3390/cancers17233723 (PMC12691007; doi:10.3390/cancers17233723)
Supplement: Supplementary file 1 [file cancers-17-03723-s001.zip › Uncropped Western Blot Images.pdf]

Figure 3B

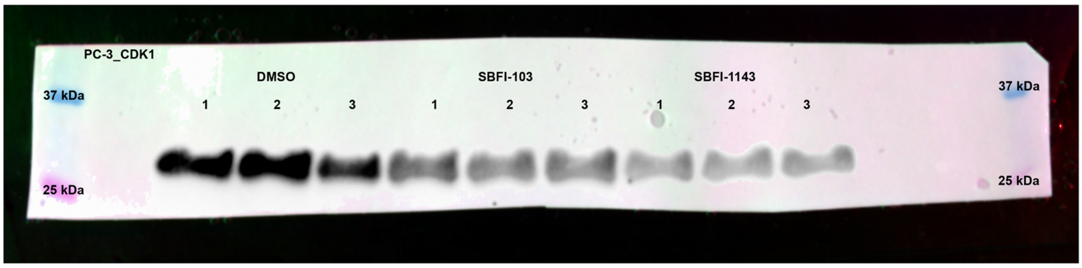

PC-3_CDK1

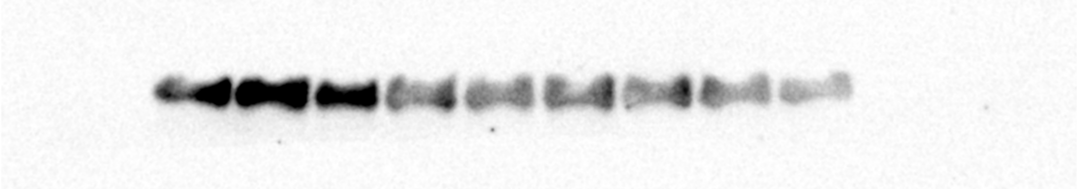

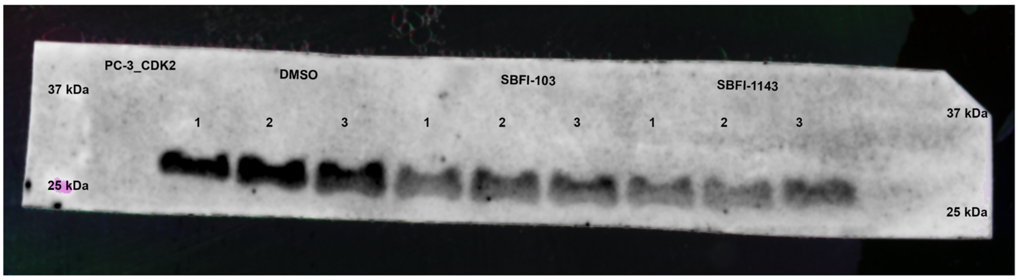

PC-3_CDK2

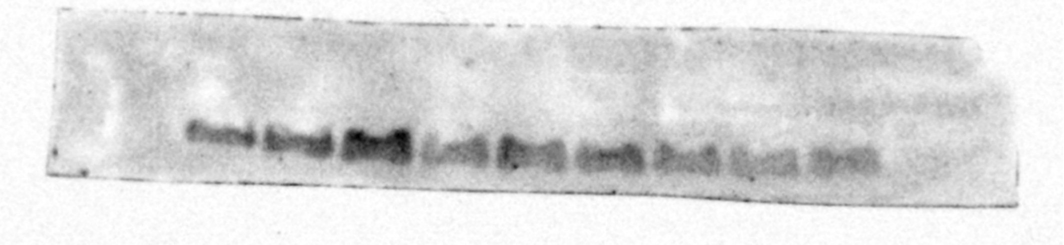

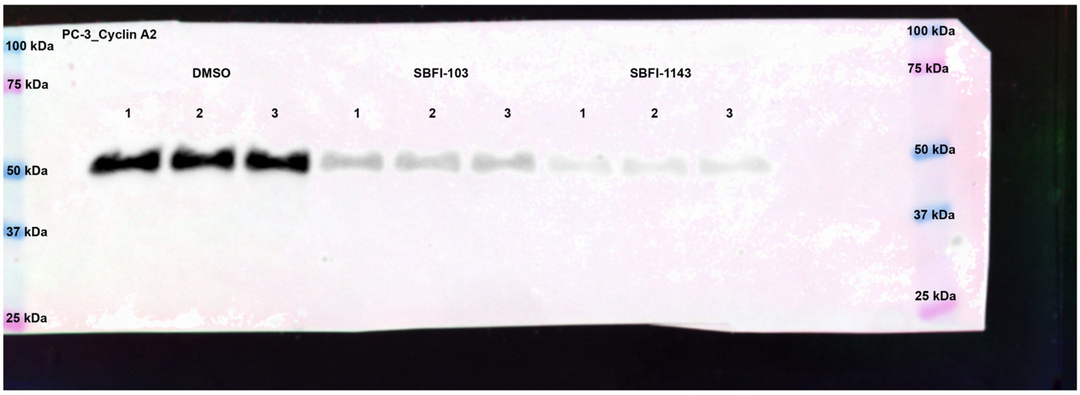

PC-3_Cyclin A2

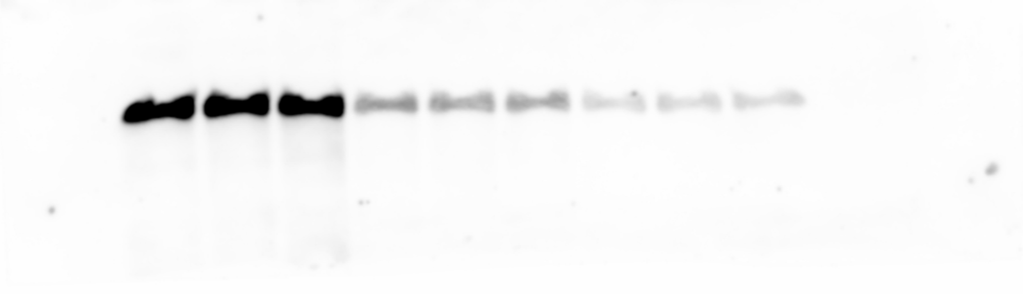

Figure 3B

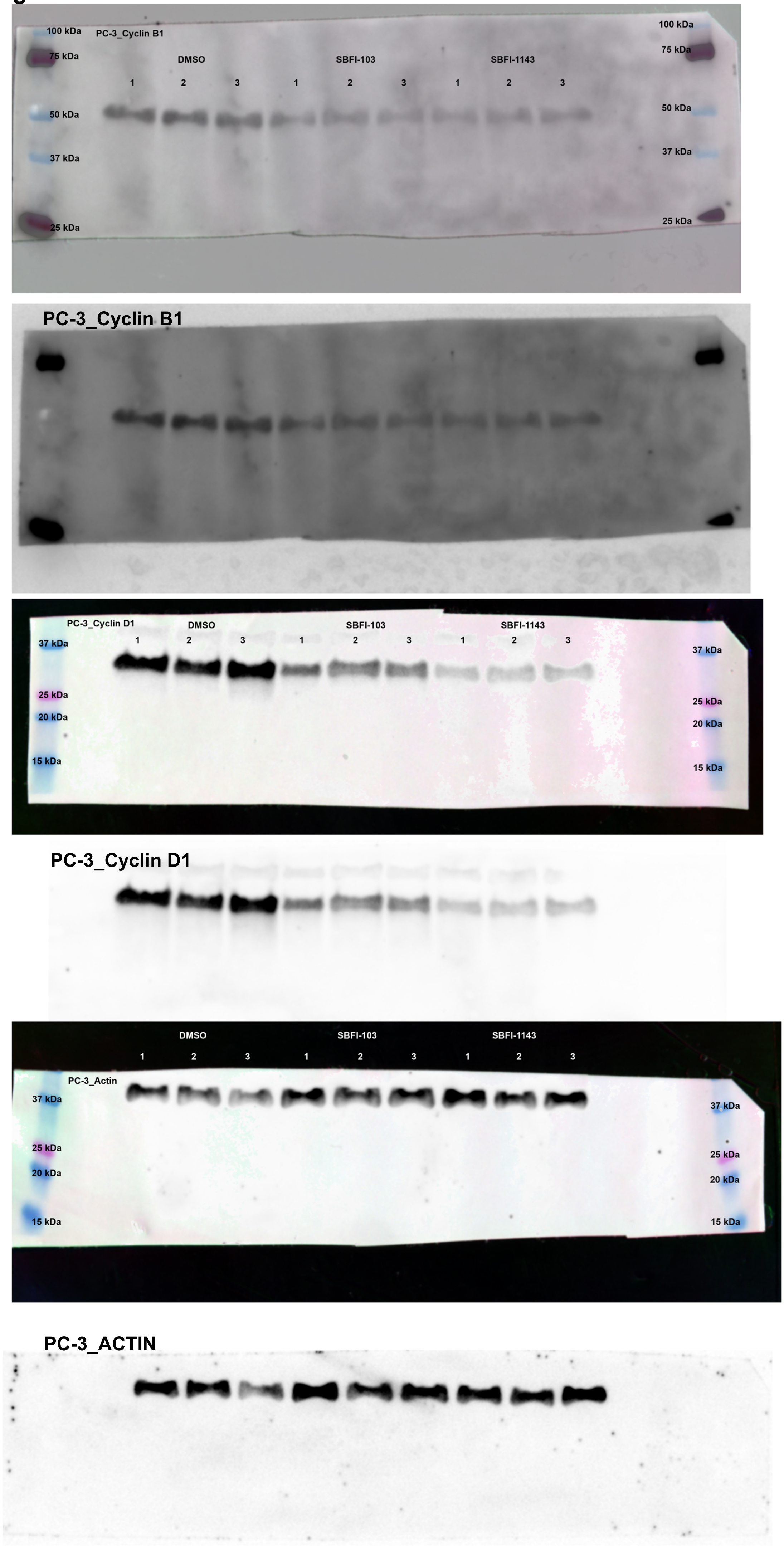

PC-3_Cyclin B1
100 kDa
75 kDa
50 kDa
37 kDa
25 kDa
DMSO
SBFI-103
SBFI-1143
1 2 3 1 2 3 1 2 3
PC-3_Cyclin B1
PC-3_Cyclin D1
37 kDa
25 kDa
20 kDa
15 kDa
DMSO
SBFI-103
SBFI-1143
1 2 3 1 2 3 1 2 3
PC-3_Cyclin D1
DMSO
SBFI-103
SBFI-1143
1 2 3 1 2 3 1 2 3
PC-3_Actin
37 kDa
25 kDa
20 kDa
15 kDa
PC-3_ACTIN

Figure 3D

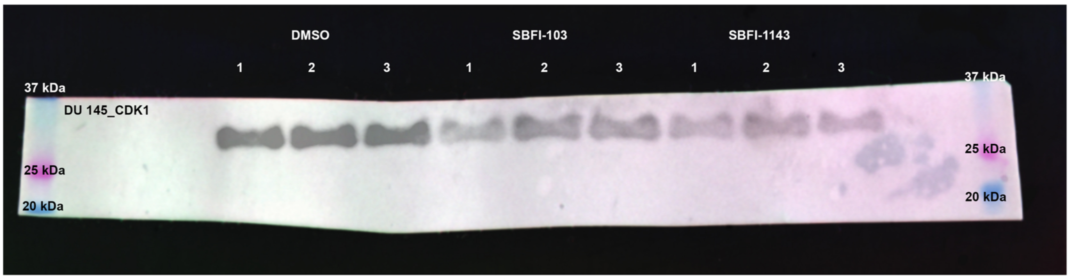

DU 145_CDK1

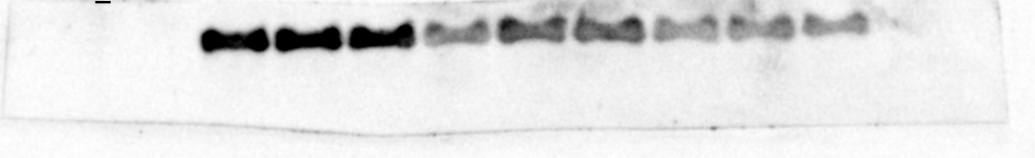

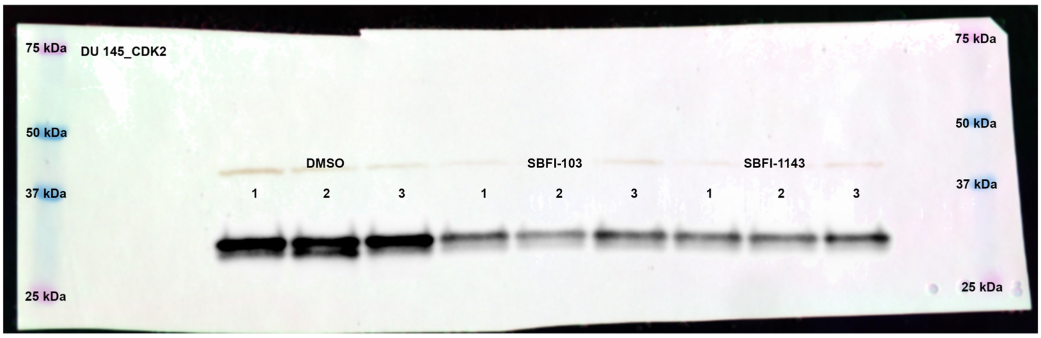

DU 145_CDK2

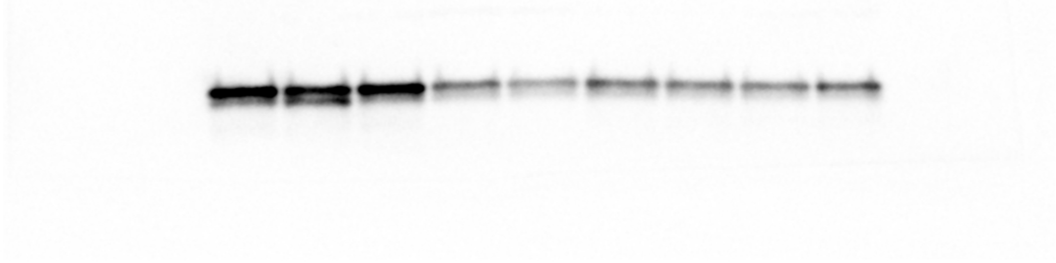

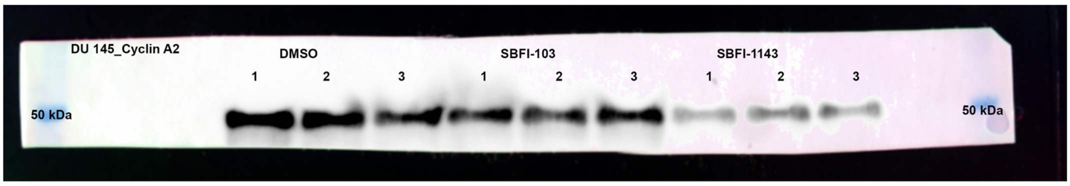

DU 145_Cyclin A2

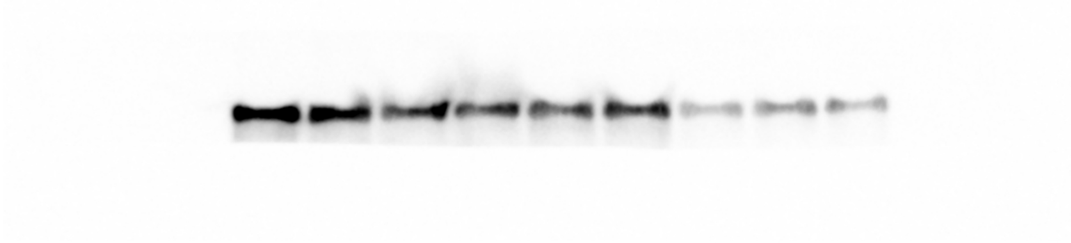

Figure 3D

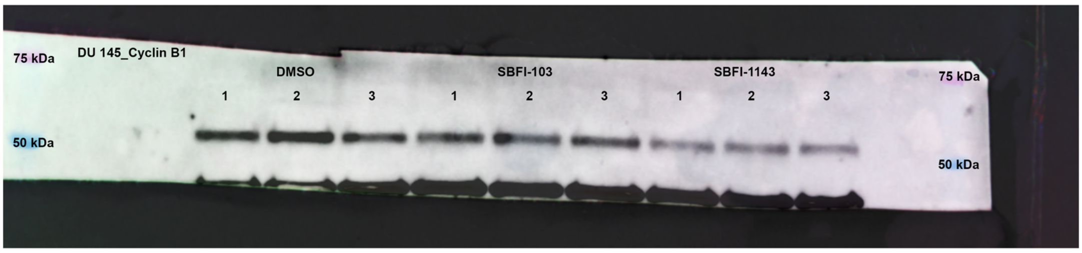

DU 145_Cyclin B1

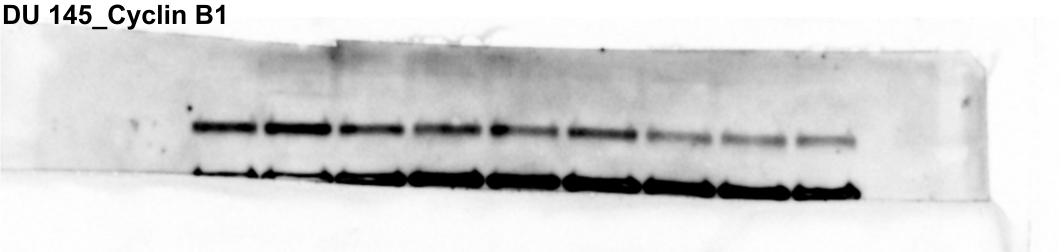

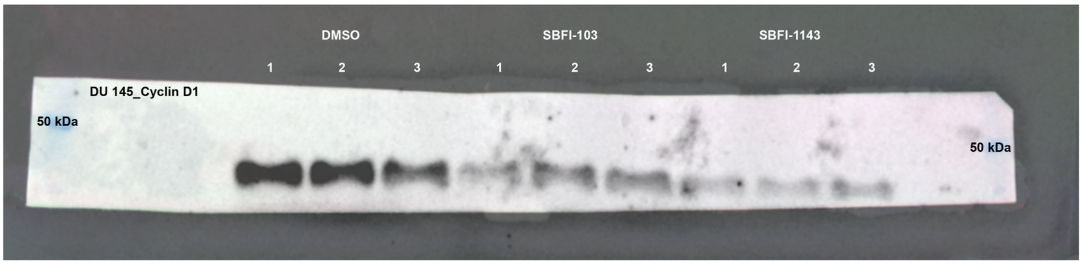

DU 145_Cyclin D1

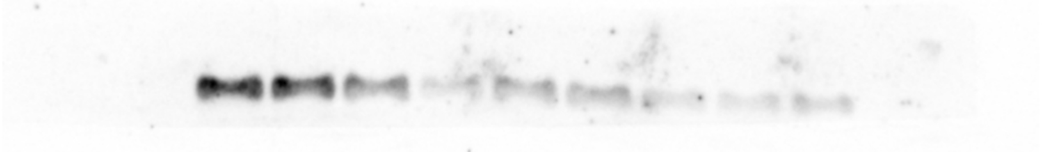

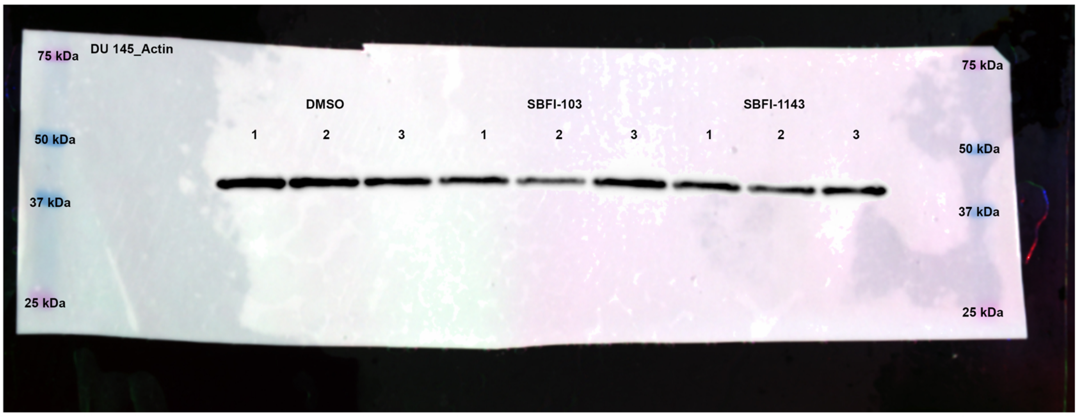

DU 145_ACTIN

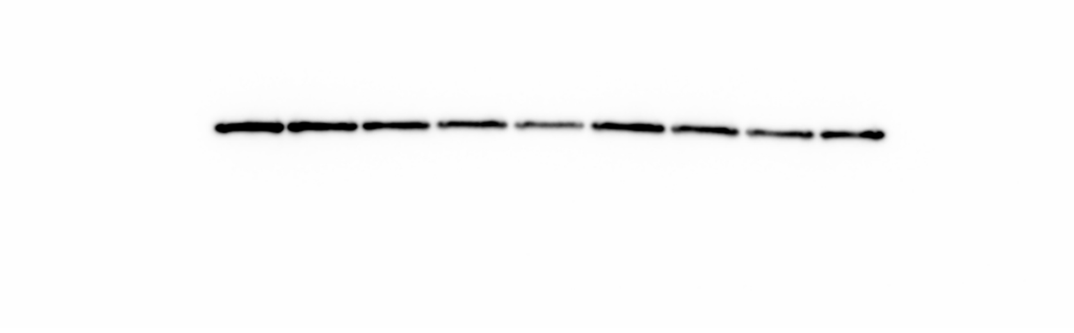

Figure 3F

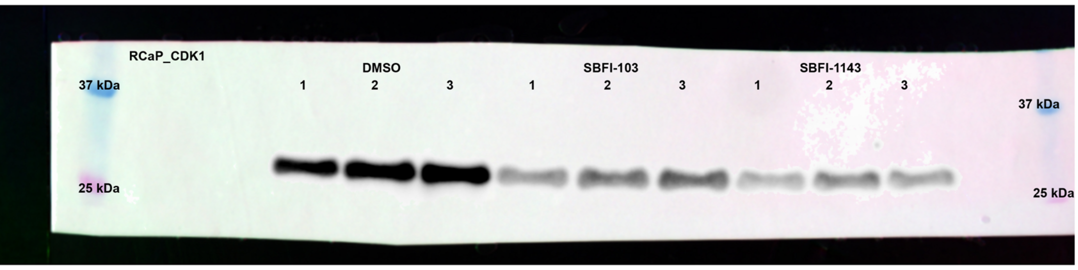

RCaP_CDK1

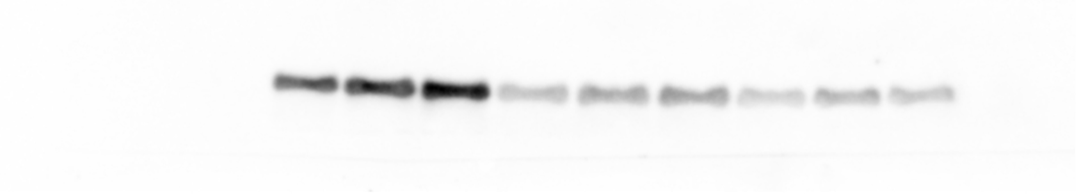

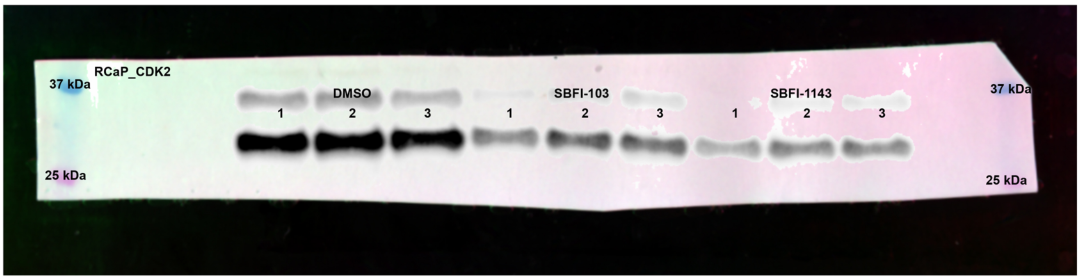

RCaP_CDK2

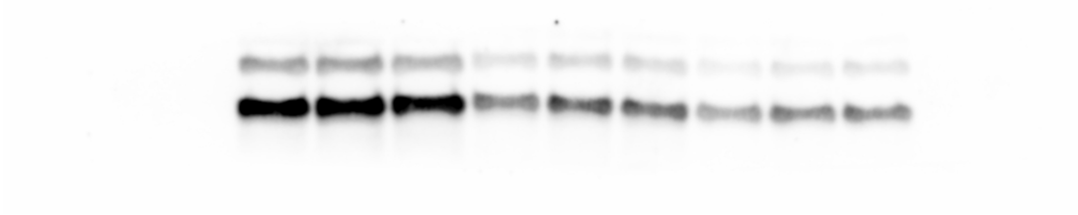

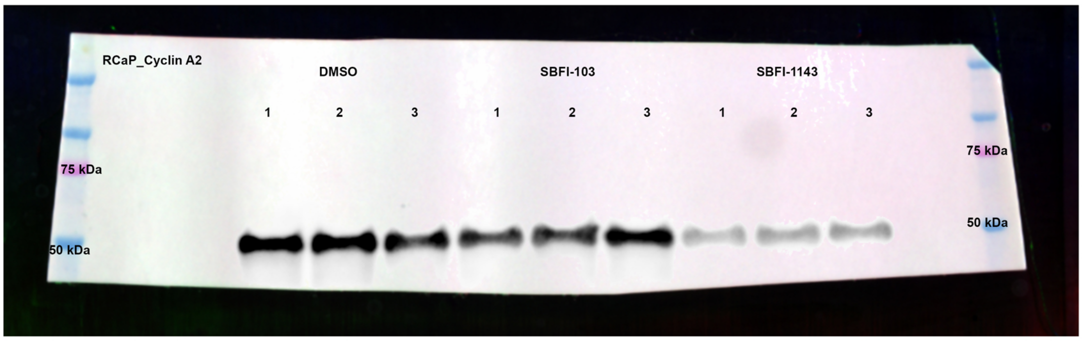

RCaP_Cyclin A2

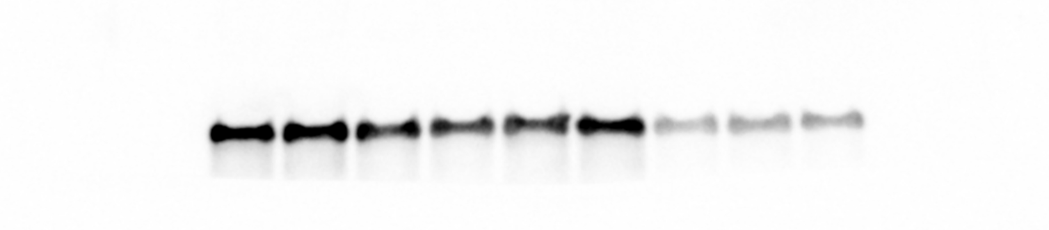

Figure 3F

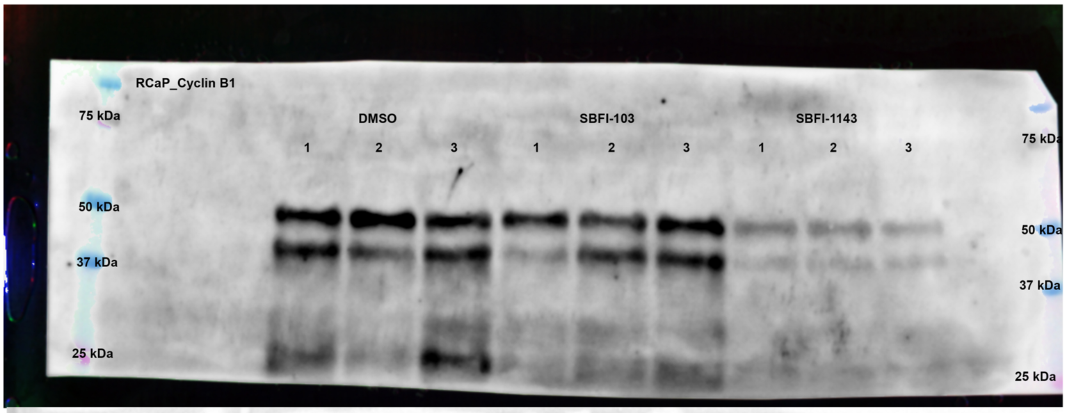
RCaP_Cyclin B1
75 kDa
DMSO
SBFI-103
SBFI-1143
1 2 3 1 2 3 1 2 3
75 kDa
50 kDa
37 kDa
25 kDa
50 kDa
37 kDa
25 kDa

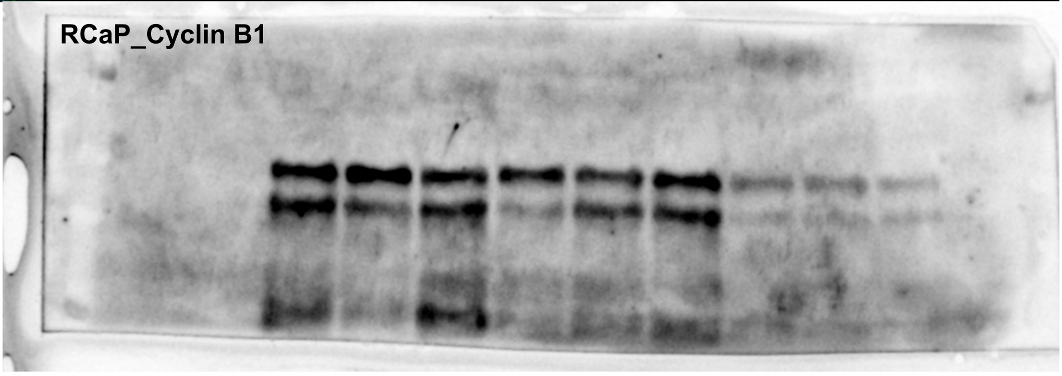
RCaP_Cyclin B1

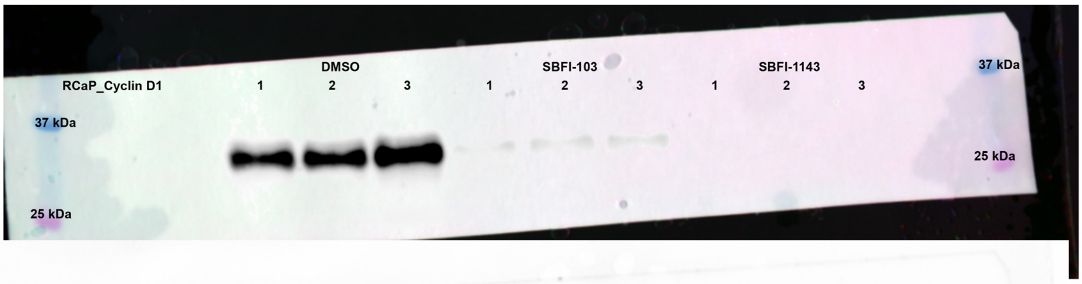
DMSO
SBFI-103
SBFI-1143
RCaP_Cyclin D1
1 2 3 1 2 3 1 2 3
37 kDa
37 kDa
25 kDa
25 kDa

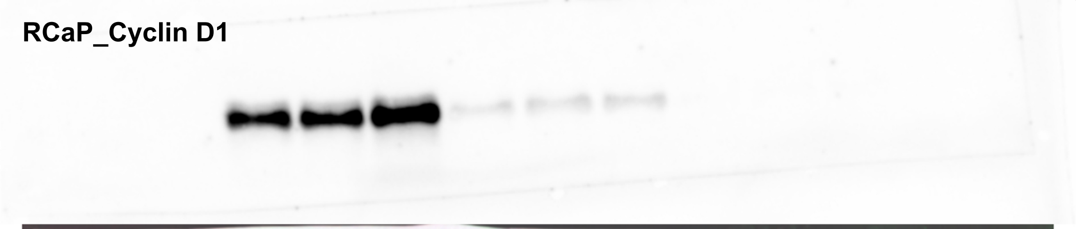
RCaP_Cyclin D1

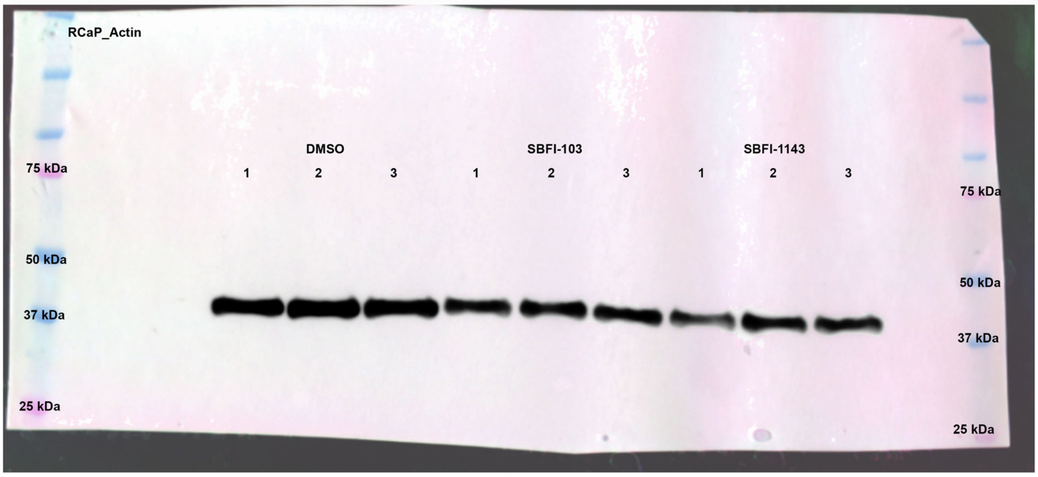
RCaP_Actin
DMSO
SBFI-103
SBFI-1143
75 kDa
1 2 3 1 2 3 1 2 3
75 kDa
50 kDa
50 kDa
37 kDa
37 kDa
25 kDa
25 kDa

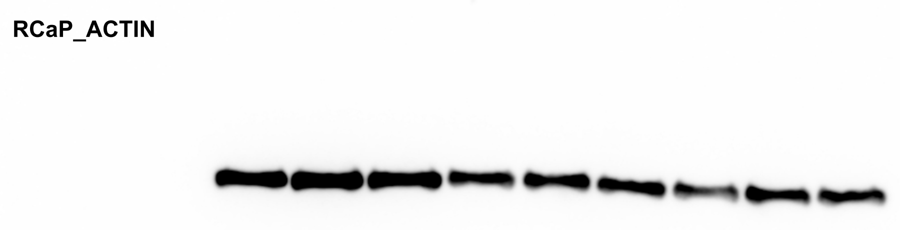
RCaP_ACTIN
